# Novel Selective Cardiac Myosin-Targeted Inhibitors Alleviate Myocardial Ischaemia–Reperfusion Injury

**DOI:** 10.1007/s10557-024-07663-0

**Published:** 2025-01-04

**Authors:** Nur Liyana Mohammed Yusof, Derek M. Yellon, Sean M. Davidson

**Affiliations:** 1 The Hatter Cardiovascular Institute, University College London, 67 Chenies Mews, London, WC1E 6HX UK; 2 Department of Pharmacology, Faculty of Medicine, Universiti Kebangsaan Malaysia, Jalan Yaacob Latif, Bandar Tun Razak, 56000 Cheras, Kuala Lumpur, Malaysia

**Keywords:** Myocardial infarction, Ischaemia, Reperfusion, Hypercontracture, Mavacamten, Aficamten

## Abstract

**Purpose:**

Reperfusion of the ischaemic heart is essential to limit myocardial infarction. However, reperfusion can cause cardiomyocyte hypercontracture. Recently, cardiac myosin-targeted inhibitors (CMIs), such as Mavacamten (MYK-461) and Aficamten (CK-274), have been developed to treat patients with cardiac hypercontractility. These CMIs are well tolerated and safe in clinical trials. We hypothesised that, by limiting hypercontraction, CMIs may reduce hypercontracture and protect hearts in the setting of ischaemia and reperfusion (IR).

**Methods:**

We investigated the ability of MYK-461 and CK-274 to inhibit hypercontracture of adult rat cardiomyocytes (ARVC) *in vitro* following ATP depletion. A suitable dose of CMIs for subsequent *in vivo* IR studies was identified using cardiac echocardiography of healthy male Sprague Dawley rats. Rats were anaesthetized and subject to coronary artery ligation for 30 min followed by 2 h of reperfusion. Prior to reperfusion, CMI or vehicle was administered intraperitoneally. Ischaemic preconditioning (IPC) was used as a positive control group. Infarct size was assessed by tetrazolium chloride staining and extent of hypercontracture was assessed by histological staining.

**Results:**

Treatment with CMIs inhibited ARVC hypercontracture *in vitro*. MYK-461 (2 mg/kg) and CK-274 (0.5 mg/kg to 2 mg/kg) significantly reduced infarct size vs. vehicle. IR caused extensive contraction band necrosis, which was reduced significantly by IPC but not by CMIs, likely due to assay limitations. GDC-0326, an inhibitor of PI3Kα, abrogated CK-274-mediated protection following IR injury. GDC-0326 reduced phosphorylation of AKT when administered together with CK-274.

**Conclusion:**

This study identifies CMIs as novel cardioprotective agents in the setting of IR injury.

**Supplementary Information:**

The online version contains supplementary material available at 10.1007/s10557-024-07663-0.

## Introduction

Infarct size is the principal determinant influencing the prognosis among patients diagnosed with acute myocardial infarction (AMI) [1]. Reimer et al. described the concept of myocardial injury as a “wavefront phenomenon”, establishing a strong relationship between infarct size and the duration of myocardial ischaemia [2]. The resumption of blood circulation to an ischaemic territory is important for maintaining tissue viability, yet it concurrently triggers multiple deleterious events known as lethal reperfusion injury [3]. Upon reperfusion, the restoration of ATP levels during ongoing cytosolic Ca^2+^ overload causes cardiomyocytes to hypercontract, which increases the likelihood of irreversible sarcolemmal rupture and cell death [4]. Hypercontracted cardiomyocytes appear as excessively shortened cells due to intense and sustained contraction. During reperfusion, hypercontracture of cardiomyocytes becomes apparent through the manifestation of contraction band necrosis (CBN) in histological sections of the myocardium. This phenomenon is characterised by the presence of distinct contraction bands, which are indicative of intense and sustained cellular contraction [5]. CBN constitutes a substantial portion of cellular death during myocardial ischaemia–reperfusion (IR) injury.

While cardiomyocyte injury plays a crucial role in IR injury and significantly contributes to long-term mortality and heart failure, there are no specific treatments in clinical use that target it [6, 7]. The contribution of cardiomyocyte hypercontracture to IR injury has been known for decades [8] and experimental evidence has demonstrated that preventing hypercontracture reduces infarct size. For instance, drugs such as 2,3-butanedione monoxime (BDM), which inhibit myofibrils, block hypercontraction in isolated perfused hearts and significantly reduce the final infarct size [9]. Unfortunately, its use in humans is not feasible due to toxicity [10]. Hypercontracture can also be inhibited, and infarct size reduced, by lowering pH during reperfusion, but, this approach is also not readily translatable [11]. Therefore, the identification of effective therapeutic strategies for mitigating hypercontracture-related damage remains an important and unmet need in the management of IR injury. There is an urgent need for novel strategies targeting hypercontracture and reducing infarct size following IR injury, alongside conventional clinical interventions like thrombolysis or immediate PCI.

Recently, a paradigm shift has occurred in the treatment of patients with hypertrophic cardiomyopathy (HCM), as selective cardiac myosin-targeted inhibitors (CMIs) have been demonstrated to reduce excessive myocardial contractility and improve heart failure symptoms in these patients [12–14]. Mavacamten (MYK-461) was the first in its class of selective CMIs that directly bind cardiac myosin heavy chain and decrease ATPase activity, and was shown to alleviate excessive cardiac contractility and impaired diastolic filling of the heart [15]. In the EXPLORER-HCM trial, Mavacamten was not only safe and exceptionally well tolerated, but also improved exercise capacity and NYHA heart failure class in patients with HCM [12, 16]. Aficamten (CK-274) is another CMI that works similarly to MYK-461 but binds to a different allosteric site [17, 18]. CK-274 has a shorter life compared to MYK-461 and appears to have a wider therapeutic window [18]. In the phase-2 REDWOOD-HCM trial in obstructive HCM patients, CK-274 was also well tolerated and showed an improvement in heart failure symptoms in the majority of patients, also decreasing levels of plasma NT-proBNP [13].

Given the success of CMIs at reducing excessive contractility in HCM patients, we hypothesized that they may also be beneficial in the setting of IR, where they may be able to limit the occurrence of severe hypercontracture, thereby increasing the recovery and viability of cardiomyocytes. An important factor to consider was that the CMIs should be used at a concentration that does not impair the normal contractile function of the heart. Initial studies were therefore conducted to ascertain the appropriate concentration of CMIs to use in rats. The current study sought to investigate the repurposing of the CMIs MYK-461 and CK-274, to reduce IR-induced hypercontracture in rats, which in turn would limit infarct size.

It is well established that activation of the PI3K/AKT kinase signalling pathway can protect the rodent heart from IR injury [19, 20]. As such, PI3K plays a key role in the so-called “RISK” (Reperfusion Injury Salvage Kinase) pathway. Furthermore, we recently showed that activation of the alpha isoform of PI3K (PI3Kα) is required for interventions such as ischaemic preconditioning (IPC) to protect the rodent heart from IR injury [21–23]. To maximise this protection, it is crucial to identify additional non-RISK pathways that could have an additive effect with IPC [24–26]. Therefore, if CMIs are shown to be cardioprotective, it would be important to establish whether these CMIs require activation of PI3K/AKT pathway. Thus, in the last part of this study, the protective effect of the CMI, CK-274, was investigated in the presence of a PI3Kα inhibitor to determine if it protects the heart independently of PI3K/AKT.

## Methods

### Ethical Approval and Animal Experiments

The animal experiments were carried out in accordance with the guidelines outlined in the Animals (Scientific Procedures) Act of 1986, operating under the Project License (PP9987686), which received approval from both the Animal Welfare and Ethical Review Board of University College London (UCL) and the UK Home Office. Adult male Sprague–Dawley rats (210–290 g), bred at UCL were used in the experiments.

### Chemicals and Reagents

MYK-461 or CK-274 were obtained from MedChemExpress and most of the reagents used here were obtained from Sigma Aldrich (later acquired by Merck KGaA) unless stated otherwise.

### Isolation of Primary Adult Rat Ventricular Cardiomyocytes

Primary adult rat ventricular cardiomyocytes (ARVC) were isolated as previously described [27, 28]. Briefly, rats were anesthetised with 100 mg/kg sodium pentobarbital with 500 IU heparin intraperitoneally. Upon loss of pedal reflex activity, thoracic surgery was performed, and the heart was rapidly excised. Isolated hearts were then placed in ice-cold isolation buffer and immediately cannulated to Langendorff apparatus via the aorta retrogradely. The hearts were perfused with isolation buffer (in mM: NaCl 130; KCl 5.4; MgCl_2_ 1.4; NaH_2_PO_4_ anhydrous 0.4; HEPES 4.2; glucose 10; taurine 20; creatine 10) with pH adjusted to 7.4. The heart was then digested by perfusing with isolation buffer containing 6 mg/100 g body weight collagenase and 2 mg protease with 100 mM CaCl_2_. After digesting all tissues, cell suspensions were gradually reintroduced to 0.5 mM CaCl_2_, then 1 mM CaCl_2_, before being resuspended in medium 199 supplemented with 5 mM creatine, 2 mM carnitine, 5 mM taurine, 50 units/mL penicillin, and 50 mg/mL streptomycin. Isolated cardiomyocytes were seeded in 24-well plates coated with laminin for at least 1 h. All cells were left to incubate overnight in a tissue culture incubator at 37 °C and 5% CO_2_ before being used further.

### *Cardiomyocyte Hypercontracture Model In Vitro*

An *in vitro* cardiomyocyte hypercontracture model was used, in which ARVC were treated with 5 μm carbonyl cyanide m-chlorophenylhydrazone (CCCP) to uncouple and de-energize mitochondria, leading to ATP depletion, cytosolic Ca^2+^ overload and hypercontracture [29–31]. On the day following cell isolation, cells were pre-treated for 30 min with either vehicle, MYK-461 or CK-274 before incubation with 5 μM CCCP for a further 30 min. During incubation, time-lapse recordings of cells were made using a GXCAM-U3PRO-6.3 software on GXM inverted microscope equipped with a 4 × objective. At each time point during the video recording, the percentage of cells that had undergone contracture was calculated by video analysis using Adobe Premiere Pro. Cell contracture was defined as the time at which a cell underwent irreversible cell shortening and reached its minimal length during the recording.

### 2D Echocardiography

Outlines for the experimental designs can be found in the supplemental material (Supplemental Figures S1–S3). Rats were anaesthetised using pentobarbital sodium (60–80 mg/kg, IP). In a supine position on a temperature-controlled heated mat, a rectal thermometer probe was inserted to maintain body temperature at 37 °C. Rats were mechanically ventilated through endotracheal intubation connected to a ventilator (VentElite, Havard Apparatus) (with 100% O_2_). Before the imaging was performed, a hair removal cream (Veet) was applied to the chest to remove the fur. The right carotid artery was cannulated using a fluid-filled catheter and connected to the PowerLab system for arterial blood pressure (BP) monitoring. 2D transthoracic echocardiography was performed using a portable Vivid *i* ultrasound (GE Healthcare, Bedford, UK) based on the protocol described previously [32]. M-mode images of the left ventricle (LV) were acquired in the parasternal long-axis and short-axis view at baseline, after 10 min, and every 30 min following intraperitoneal (IP) injection of CK-274 and MYK-461 at doses of 0.5 mg/kg, 1 mg/kg, and 2 mg/kg over a 3-h duration. M-mode was used to measure the internal dimensions of the LV cavity. LV ejection fraction (LVEF) was calculated offline based on the internal dimensions of the LV at end-diastole and end-systole point using EchoPAC software (GE Healthcare). Additionally, blood pressure was continuous monitoring using a fluid-filled catheter inserted into the right carotid artery and connected to the PowerLab system.

### *Induction of Myocardial Ischaemia and Reperfusion In Vivo and Infarct Size Measurement*

We used an established non-recovery protocol of *in vivo* rat coronary artery occlusion reperfusion model [28, 33]. Male Sprague Dawley rats (210–290 g) were anaesthetised with 60–100 mg/kg sodium pentobarbital. A standard ECG and body temperature (37 °C) were monitored throughout the procedure. Tracheal intubation was performed using an intubation catheter connected to a ventilator (VentElite, Harvard). Ischaemia was induced by the ligation of the left anterior descending (LAD) artery for 30 min. This was followed by 2 h reperfusion by releasing the snare. At the end of reperfusion, the LAD artery was re-occluded, and 5% Evan’s Blue dye solution was injected into the jugular vein to demarcate the area at risk. The heart was then removed from the chest and stained with tetrazolium chloride for infarct size measurement and analysed using the methods previously described [34].

### Experimental Protocol

Outlines for the experimental designs can be found in the supplemental material (Supplemental Figures S4–7). MYK-461 and CK-274 (up to 2 mg/kg) or vehicle (mixture of 0.9% corn oil & 0.1% DMSO) was administered by intraperitoneal bolus injection, 10 min or 30 min prior to reperfusion. Ischaemic preconditioning (IPC) was administered using 2 cycles of 5 min LAD occlusion followed by 5 min reperfusion prior to index ischaemia. To determine whether MYK-461 and CK-274 were cardioprotective, MYK-461 and CK-274 were administered at 30 min and 10 min prior to reperfusion, respectively. This was to allow sufficient time for the drugs to reach equilibrium with their binding sites in the heart and was based on the time it took to see effect on LVEF.

#### Study 1

There were five groups of rats, which were all subject to IR of 30 min ischaemia followed by 2 h reperfusion, as described above. The control group received vehicle. The second group served as a positive control group and received IPC (described above) prior to ischaemia. Three treatment groups received MYK-461 at different doses (0.5 mg/kg, 1 mg/kg, and 2 mg/kg). MYK-461 or vehicle was given via IP injection 30 min prior to reperfusion. The treatments were randomised, and treatments and analyses were conducted blinded to treatment.

#### Study 2

Rats were subject to 40-min ischaemia (in order to induce larger infarcts that would be sufficient to identify potential additive benefits of IPC and MYK-461) followed by 2 h reperfusion. The control group received vehicle. IPC was administered prior to ischaemia as above. Group 3 received IPC as well as 2 mg/kg MYK-461, 30 min prior to reperfusion.

#### Study 3

All four groups were subject to 30-min ischaemia, followed by a 2-h reperfusion. The first group was designated as the control group. Three groups received CK-274 at different doses (0.5 mg/kg, 1 mg/kg, and 2 mg/kg). CK-274 or vehicle was given via IP injection 10 min prior to reperfusion.

#### Study 4

All six groups were subject to 30-min ischaemia, followed by a 2-h reperfusion. The first group was the vehicle control group. In the second group, rats were given 1 mg/kg GDC-0326 (PI3Kα inhibitor) 5 min prior to reperfusion. The third group (IPC group), served as a positive control group. This was followed by IPC with the GDC-0326 group, whereby IPC was carried out prior to ischaemia and rats were given 1 mg/kg of GDC-0326 5 min prior to reperfusion. 1 group received 0.5 mg/kg of CK-274 alone, and lastly, a combination of treatment with 0.5 mg/kg of CK-274 and 1 mg/kg of GDC-0326 was given prior to reperfusion. All vehicles (a mixture of 90% corn oil and 10% DMSO) and 10% DMSO in normal saline were given via IP, 10 and 5 min prior to reperfusion, respectively.

### Western Blot Analysis

Outlines for the study protocols for this western blot can be found in the supplemental material (Figure S8). In brief, rats underwent IPC, and the hearts were excised immediately after reperfusion onset. Rats were randomly assigned to one of the following six groups (n = 4): vehicle, 1 mg/kg GDC-0326, IPC, IPC + 1 mg/kg GDC-0326, 0.5 mg/kg CK-274, and 0.5 mg/kg CK-274 + 1 mg/kg GDC-0326. The heart was snap frozen in liquid nitrogen, and stored at −80 °C before being processed into tissue lysates using standard techniques [23]. Frozen heart tissue was weighed and homogenised in an ice-cold lysis buffer containing 100 mM Tris (Sigma), 300 mM NaCl (Sigma), 1% IGEPAL (Sigma), and 1 × EDTA (Sigma) with adjusted pH 7.4 and protease inhibitor cocktails (Thermo Scientific). After being centrifuged, the supernatants were collected and stored at −80 °C. The total protein concentrations in the samples were measured by using the BCA protein kit assay (Sigma). The samples were mixed with 4X Invitrogen Novex NuPAGE LDS sample buffer (Thermofisher Scientific) and 10% β-mercaptoethanol (Sigma). The samples were then denatured by heating them to 80 °C for 10 min. Samples containing an equal amount of protein (20 µg) were evaluated to determine protein expression of Akt (pan) (#2920, Cell Signalling) and Phospho-Akt (Ser473)( #9271, Cell Signalling). Next, using the Mini Protean III system (Bio-Rad, UK), samples were loaded onto NuPAGE Novex 10% Bis–Tris protein gels (Thermofisher Scientific, UK). Proteins were transferred onto Immobilon®-P PVDF membrane (Merck) through wet transfer in a Bio-Rad Mini Trans-Blot. The membranes were blocked for 1 h using 5% skimmed milk and incubated with primary antibodies (dilution of 1:1000) at 4 °C overnight. After overnight incubation, membranes were incubated with secondary antibodies (IRdye 680LT antibodies, for red bands, Odyssey) and (IRdye 800CW antibodies, for green bands, Odyssey). Finally, protein concentrations were determined using the Odyssey imaging system from Li-Cor Biosciences (Image Studio Lite Version 5.2).

### Histology Study

To study the impact of CMIs on histological changes following reperfusion, a new set of non-recovery in vivo IR experiments was conducted, in which a 30-min ischaemia was performed to induce MI, followed by 30-min reperfusion. One group served as a sham group. The second, control group, received vehicle. The IPC group served as a positive control. Treatment groups received 2 mg/kg MYK-461 or 2 mg/kg CK-274. Drugs or vehicle (as above) was given via IP injection 10/30 min prior to reperfusion. Supplementary Figure S9 summarises the experimental protocol in this set of experiments. At the end of the experiment, the hearts were excised, cut into longitudinal sections, and fixed in 4% paraformaldehyde and 70% ethanol for tissue processing. All the heart samples then underwent processing, embedding, and tissue sectioning. The paraffinized sections were then stained to determine the area containing hypercontracted cardiomyocytes (CBN), as elaborates in detail in the supplementary materials (Supplementary Figure S10 and S11). For the histological visualisation of heart tissue following IR injury, a phosphotungstic acid haematoxylin (PTAH) stain kit (Abcam) was used. PTAH clearly stains muscle cross-striations and fibrin blue. All the staining procedures were carried out in accordance with the manufacturer's instructions.

### Statistical Analysis

GraphPad Prism 9.5.1 was used for statistical analysis and graph production (GraphPad Software). All data are presented in the form of mean value ± SEM. Parametric data (Arterial blood pressure, AAR, IS,) were analysed using a one- or two-way ANOVA as indicated, followed by Tukey’s multiple comparison test. For the PTAH analysis, Kruskal–Wallis with Dunn’s post hoc tests was used. p < 0.05 was considered statistically significant. All image quantifications were conducted in a blinded manner to minimise potential bias during the analysis process. Investigators were blinded to the treatment before administration, and analysis was conducted blinded to treatment.

## Results

### MYK-461 and CK-274 Reduce Cardiomyocyte Hypercontracture *In Vitro*

**Fig. 1:**
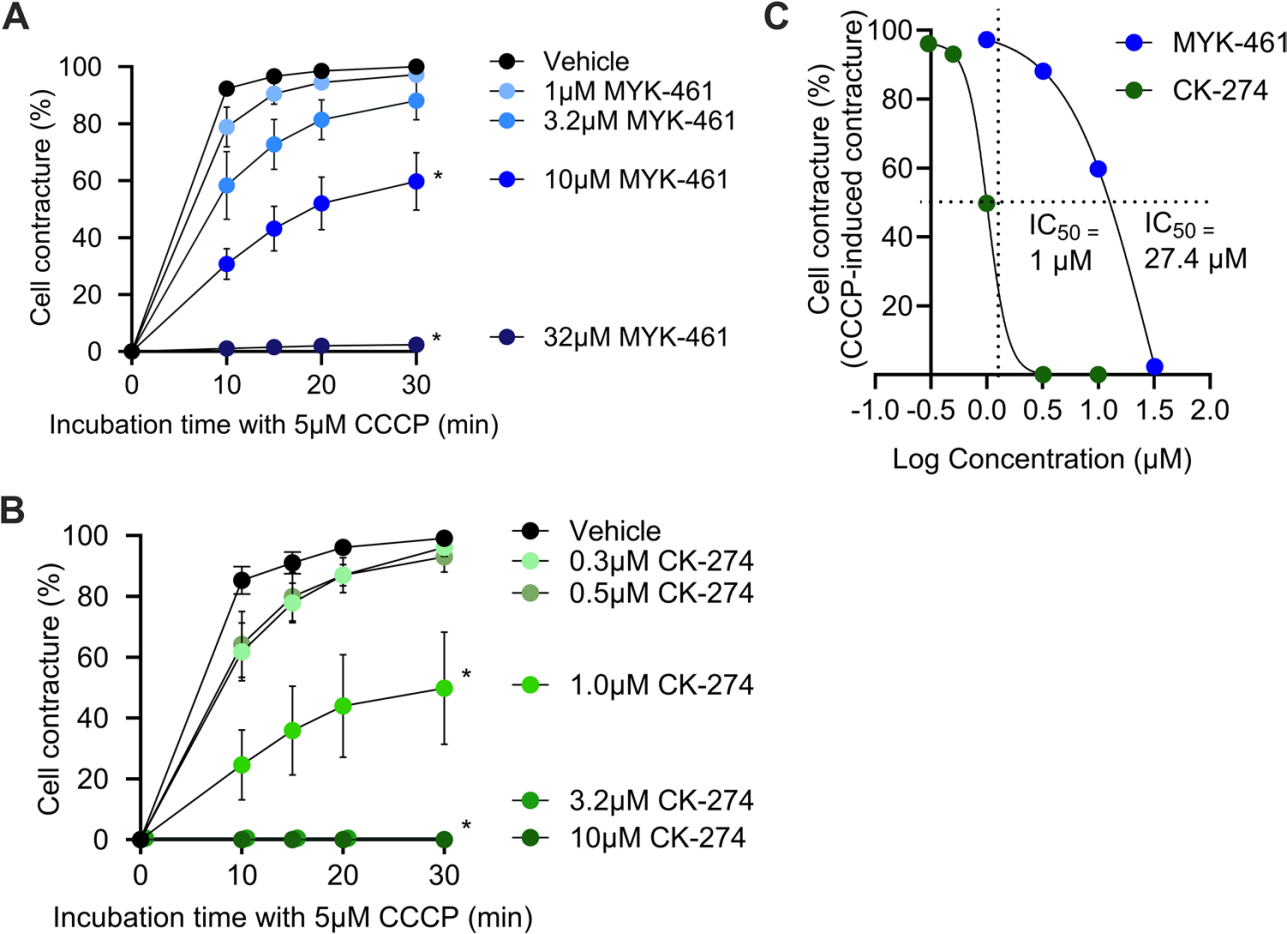
Both MYK-461 and CK-274 inhibit cardiomyocyte contracture induced by treatment with CCCP *in vitro*, with CK-274 exhibiting greater potency. Dose response of (**A**) MYK-461 and (**B**) CK-274 on the percentage of cells that underwent contracture over time, after addition of CCCP. Data is represented as mean ± SEM from n = 4–7 independent experiments, in which 200–300 cells were analysed for each treatment. (**C**) Concentration-dependent inhibitory effects of MYK-461 and CK-274 on CCCP-induced contracture vs log concentration (derived from panels A and B). The IC50 is indicated. * p < 0.05 vs time-matched vehicle, assessed using two-way ANOVA with Tukey’s post-test

ARVCs were incubated with either MYK-461 (Fig. 1A) or CK-274 (Fig. 1B) for 30 min prior to addition of 5 μM CCCP to induce hypercontracture. In the presence of 10 μM MYK-461 the cells developed significantly less contracture after 30 min (60 ± 10%) than vehicle (100%) (p < 0.05). 32 μM MYK-461 completely inhibited cell contracture, resulting in only 2 ± 2% of cells displaying contracture (Fig. 1A**)**. CK-274 was more potent, significantly inhibiting cell contracture by 50 ± 19% at only 1 μM (p < 0.05) compared to the vehicle. 3.2 μM and 10 μM CK-274 fully inhibited contracture (Fig. 1 B). A best-fit curve (Fig. 1C**)** identified the IC_50_ for CK-274 in this cellular assay as 1 μM, compared to IC_50_ of 27.4 μM for MYK-461.

### *Dose–Response of MYK-461 and CK-274 on LV Function In Vivo*

**Fig. 2:**
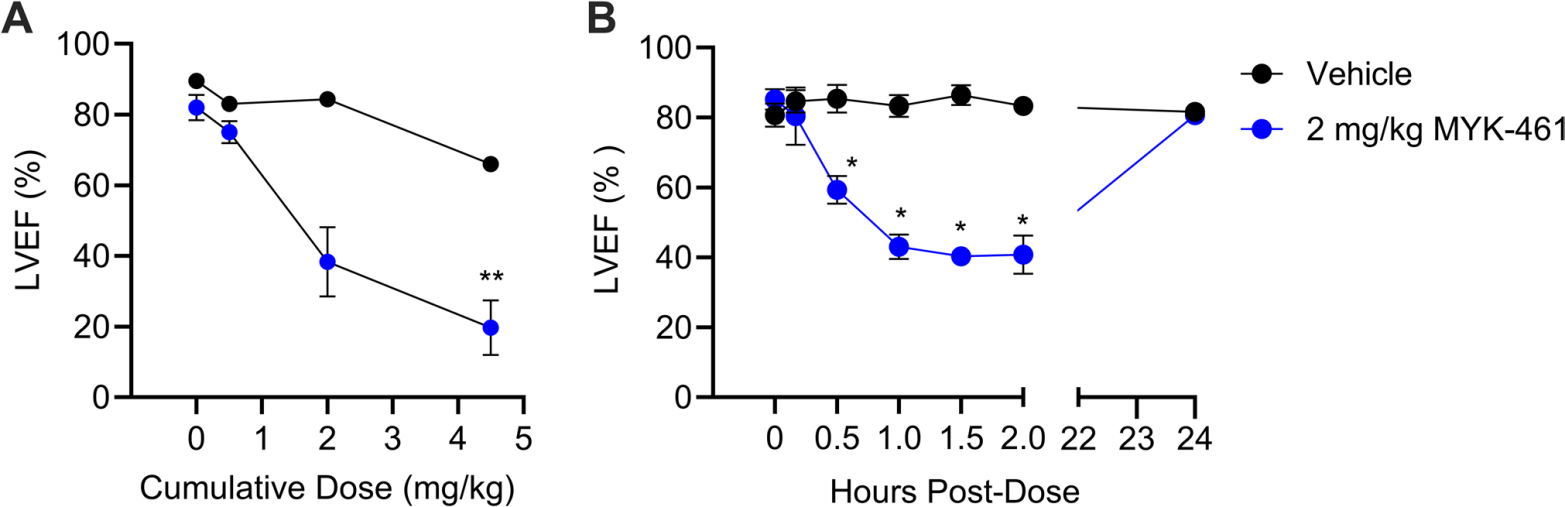
Left ventricular ejection fraction (LVEF) is significantly reduced in rats by 2 mg/kg MYK-461 i.p., although this is transient and LVEF returns to baseline by 24 h. (**A**) The dose response effect of MYK-461 on LVEF in anaesthetized rats (n = 4, vs 1 representative vehicle control). (**B**) LVEF over time following a single dose of 2 mg/kg MYK-461 or vehicle (n = 5/group) i.p. Data is represented as mean ± SEM, * p < 0.05, ** P < 0.01, assessed using one-way, repeated-measures ANOVA with Dunnett’s multiple comparisons test to compare to baseline (A) or two-way ANOVA with Tukey’s post-test (B)

In a pilot dose-ranging experiment, echocardiography was used to measure LVEF in healthy rats following the administration of an increasing dose of MYK-461 (Fig. 2). 2 mg/kg of MYK-461 caused a non-significant decrease in LVEF from 82 to 40% and this decreased further and significantly (p < 0.05) to 20% after 4.5 mg/kg MYK-461 (Fig. 2A). From this, we identified 2 mg/kg as the maximum dose to be used in subsequent studies, as it did not appear to significantly affect LV function.

In the second study, the LV function was monitored following the administration of 2 mg/kg MYK-461 in healthy rats. Changes in LVEF were monitored 24-h post-administration of MYK-46. Figure 2B demonstrates a significant reduction in LVEF within the MYK-461 treated group, with the greatest decrease observed 1—2 h after administration (p < 0.05), where it decreased to 40%—50% relative to the vehicle treatment. Importantly, however, LVEF returned to baseline by 24 h.

**Fig. 3:**
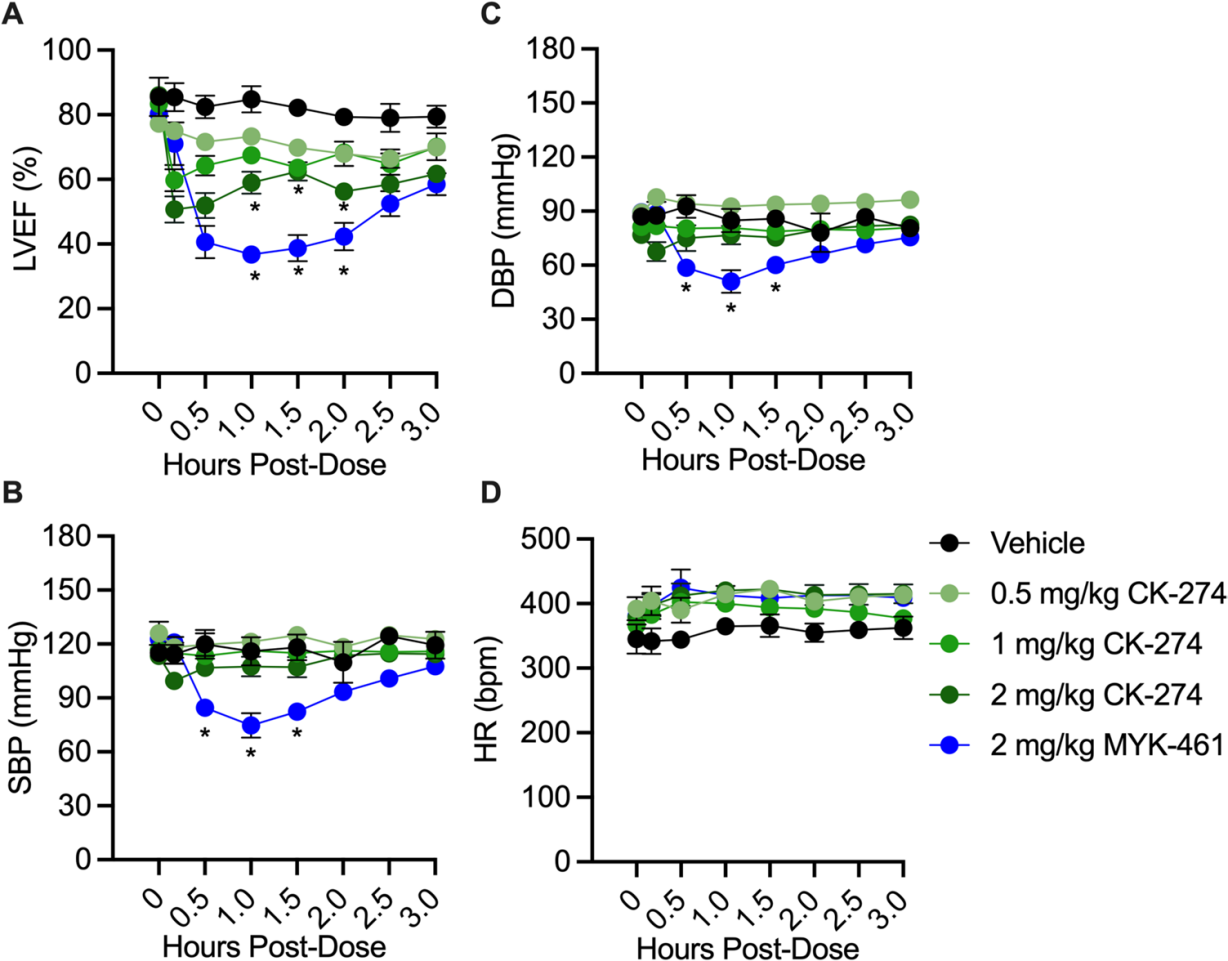
A comparison of the time-course of haemodynamic effects following a single dose of CK-274 or MYK-461 in rats. (**A**) Left ventricular ejection fraction (LVEF), (**B**) systolic blood pressure (SBP), (**C**) diastolic blood pressure (DBP) and (**D**) heart rate (HR) was assessed following single doses of CK-274 or MYK-461 (0.5 to 2 mg/kg i.p.) to anaesthetised rats. Data was represented as mean ± SEM, * p < 0.05 vs. time-matched vehicle, assessed using two-way ANOVA with Tukey’s multiple comparisons (n = 3–6/group)

In contrast to the effect of 2 mg/kg MYK-461 on LV function, CK-274 had a much less pronounced effect at 2 mg/kg or 1 mg/kg, and no significant effect at all at 0.5 mg/kg (Fig. 3A). The reductions in LVEF remained significant (p < 0.05) in both the 2 mg/kg CK-274 group and the MYK-461 group at the end of 3-h experiment. 2 mg/kg MYK-461 also caused a significant decrease in SBP and DBP from 30 to 90 min post-administration. The heart rate remained stable across all experimental groups, as shown in Fig. 3B-D.

### *Infarct Size Following MYK-461 and CK-274 Administration In Vivo*

**Fig. 4:**
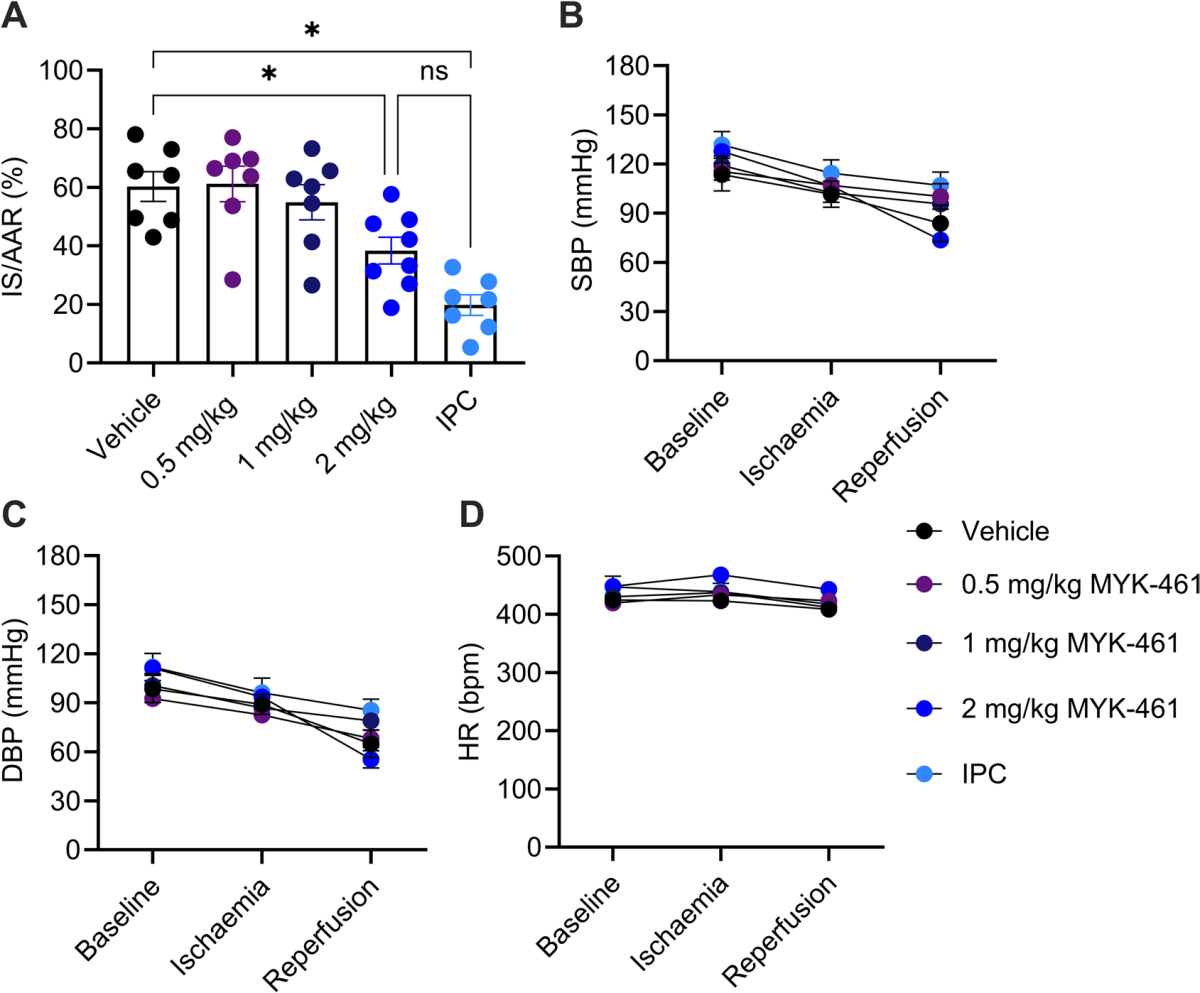
MYK-461 reduces infarct size following IR in rats. (**A**) The infarct size (IS) was expressed as a percentage of the area at risk (AAR). (**B**) Systolic blood pressure (SBP), (**C**) diastolic blood pressure (DBP) and (**D**) heart rate (HR) was monitored throughout the experiment. Data was represented as mean ± SEM value, * p < 0.05 assessed using one-way ANOVA with Tukey’s post-test n = 7–8 for each group, ns: non-significant differences, IPC:ischaemic preconditioning

The quantification of the infarct size as percentage of area at risk (AAR), following 30 min ischaemia and 2 h reperfusion, is shown in Fig. 4A. AAR as a percentage of the LV was similar between the groups, indicating procedural consistency (Supplementary Figure S12). 0.5 mg/kg and 1 mg/kg MYK-461 did not significantly alter infarct size, but 2 mg/kg MYK-461 was cardioprotective, reducing infarct size (38 ± 5%) compared to the vehicle group (60 ± 5%) (p < 0.05) (Fig. 4A). IPC (20 ± 4%) served as a positive control and demonstrated significant myocardial protection compared to the vehicle group (p < 0.05). SBP, DBP, and HR were continuously monitored throughout the experiment and no differences were observed between groups (Fig. 4B-D).

**Fig. 5:**
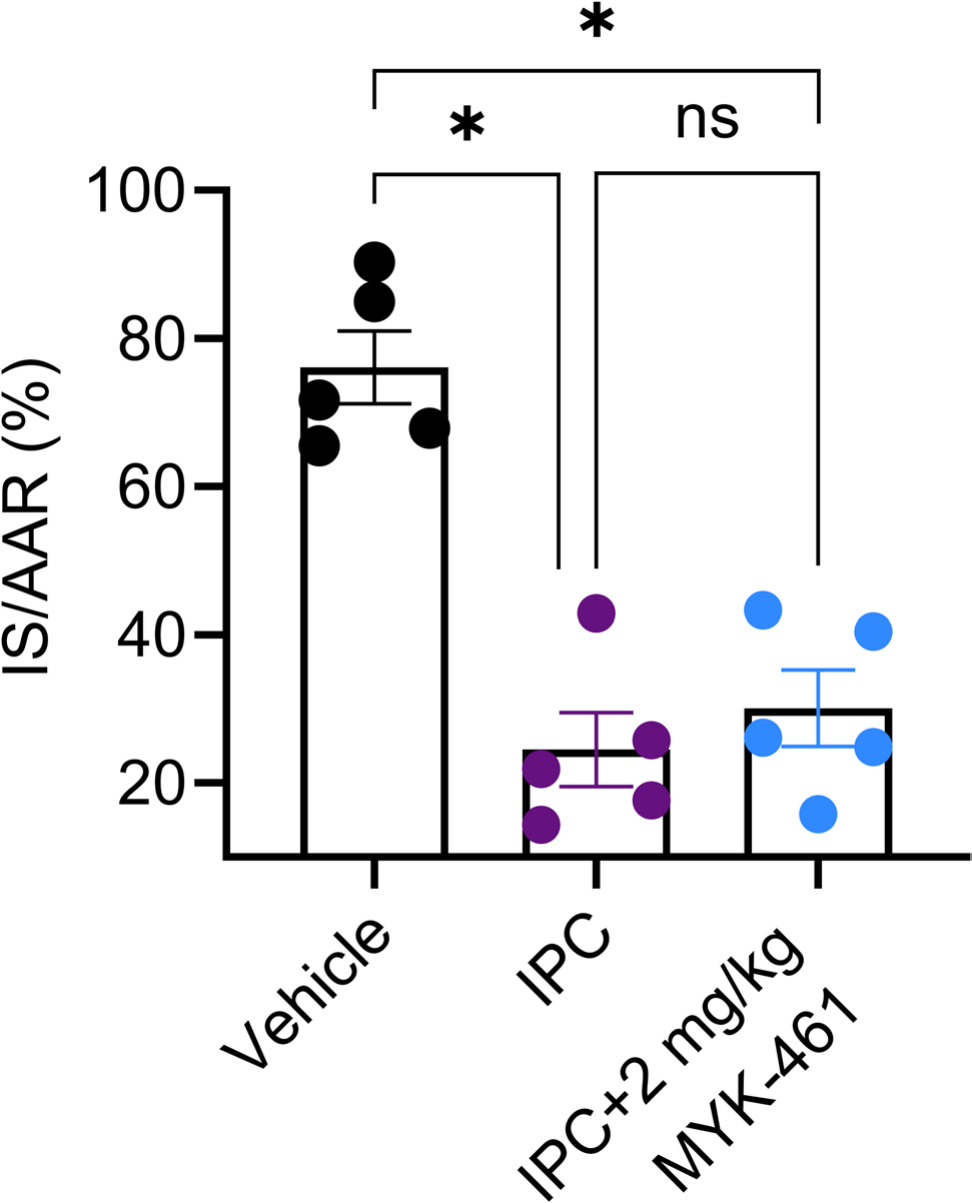
The combination of 2 mg/kg MYK-461 with ischaemic preconditioning (IPC) did not reduce the infarct size more than IPC alone, after 40 min ischaemia and 2 h reperfusion. The infarct size (IS) was expressed as a percentage of the area at risk (AAR). Data is represented as mean ± SEM value, * p < 0.05 assessed using one-way ANOVA with Tukey’s multiple comparisons with n = 5 for each group, ns: non-significant differences

Since 2 mg/kg of MYK-461 reduced infarct size following IR, an experiment was conducted to determine whether MYK-461 could provide further protection on top of IPC. For this experiment, the duration of ischaemia was increased from 30 to 40 min. However, while both the IPC (25 ± 5%) and MYK-461 + IPC (30 ± 5%) groups exhibited a significant decrease in infarct size (p < 0.05) compared to the vehicle group (76 ± 5%), there was no difference between the two treated groups (Fig. 5A). AAR was similar between the groups (Supplementary Figure S13-S15).

**Fig. 6:**
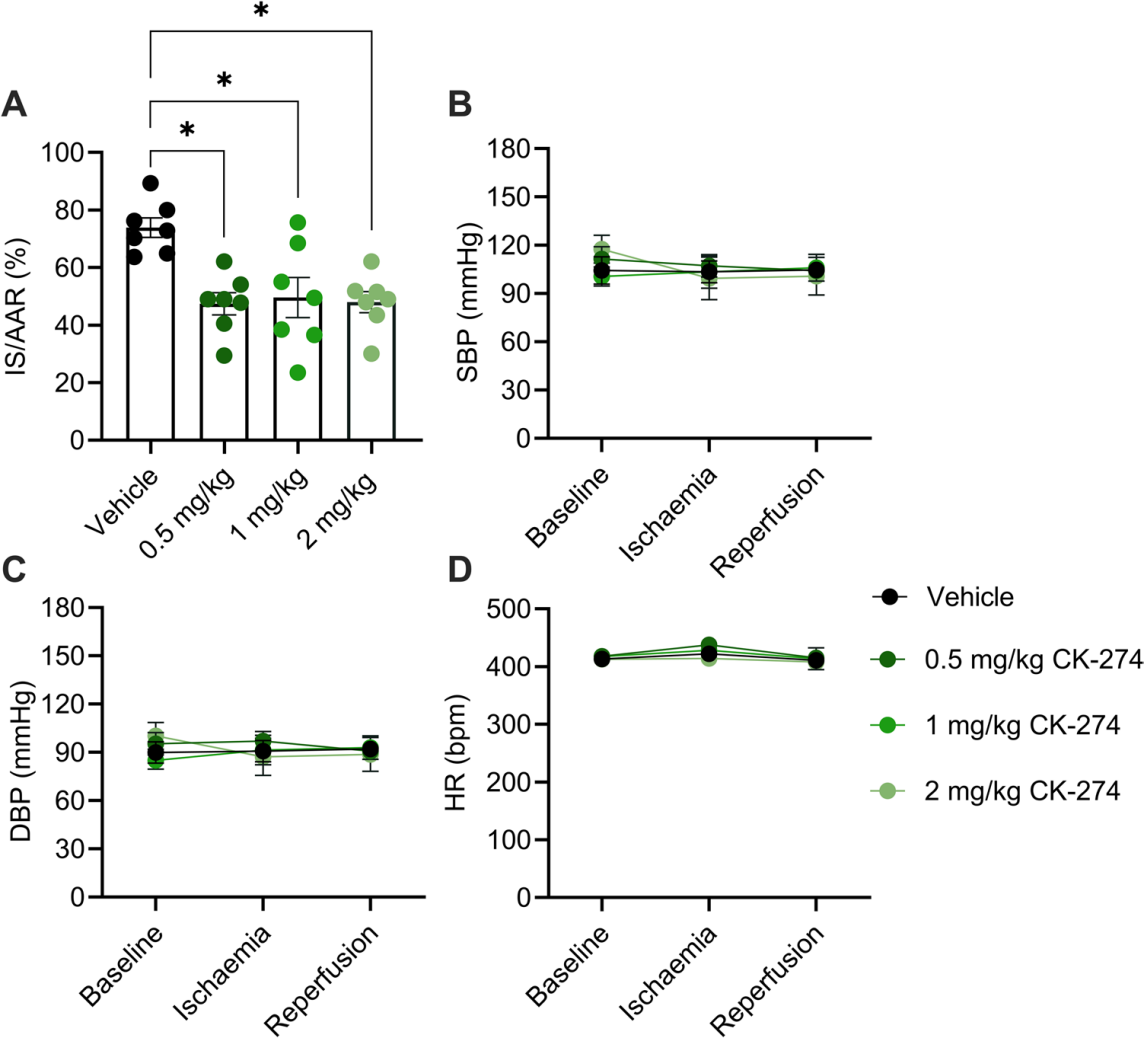
CK-274 reduces infarct size following IR in rats. (**A**) Effect of CK-274 on infarct size in rats subject to IR. (**B**) systolic blood pressure (SBP), (**C**) diastolic blood pressure (DBP) and (**D**) heart rate (HR) was monitored throughout the experiment. Data is represented as mean ± SEM, * p < 0.05 assessed using one-way ANOVA with Tukey’s multiple comparisons with n = 7 for each group

We further investigate the ability of CK-274 to reduce IR injury, and found that at all tested dosed, infarct size was significantly reduced (0.5 mg/kg: 47 ± 4%; 1 mg/kg: 50 ± 7%; 2 mg/kg: 48 ± 4%) compared to the vehicle group (74 ± 3%) (p < 0.01) **(**Fig. 6A**)**. The blood pressure and the heart rate remained stable across all experimental groups (Fig. 6C-E).

### PI3Kα Mediates Protection by CK-274 against Myocardial IR Injury

**Fig. 7:**
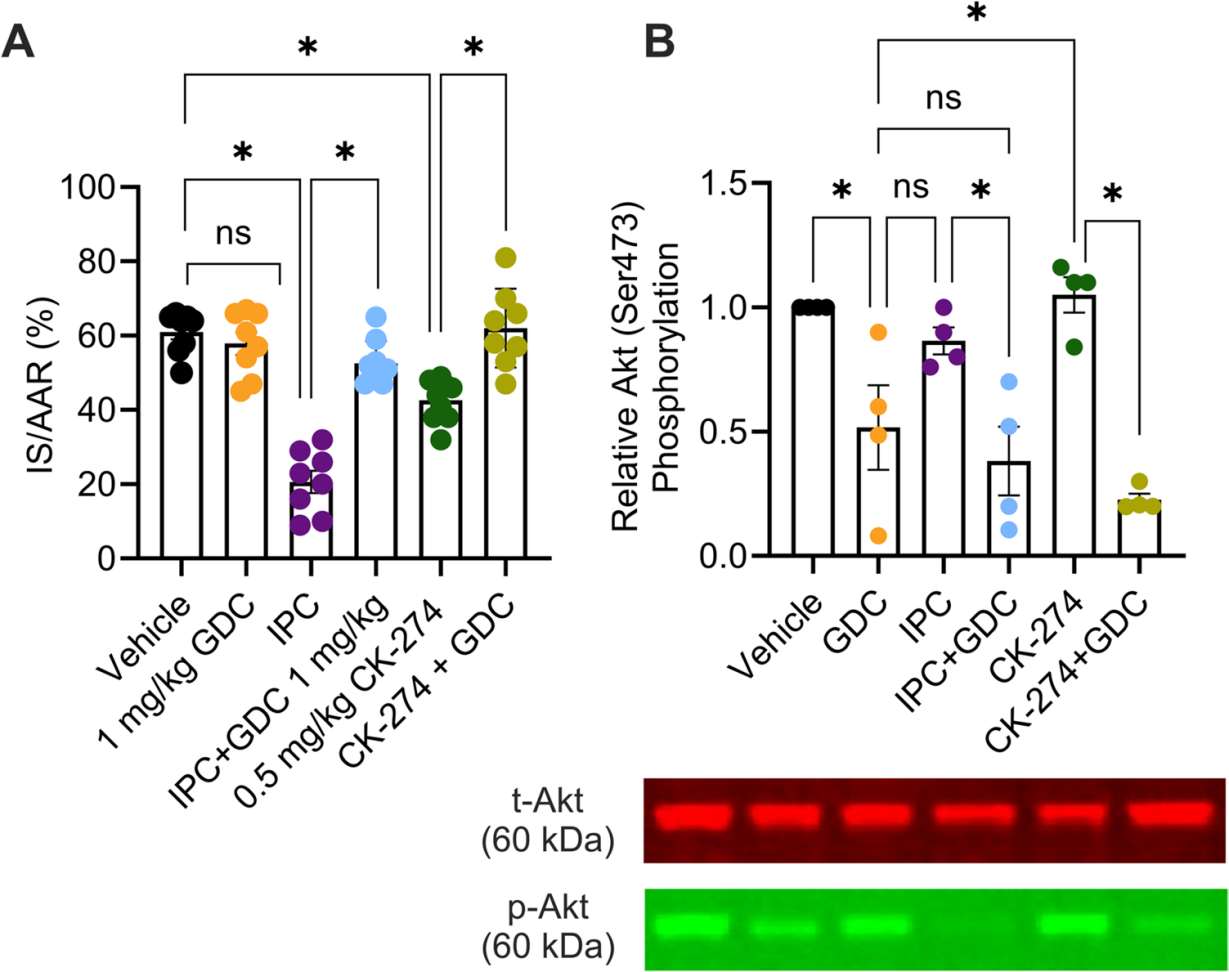
The selective PI3Kα inhibitor, GDC-0326 prevents infarct size reduction with CK-274 treatments following myocardial IR injury. (**A**) The infarct size is expressed as a percentage of the AAR. 1 mg/kg of GDC-0326 (GDC) abolished the cardioprotective effects of 0.5 mg/kg of CK-274, resulting in an infarct size comparable to that of the vehicle group (n = 8–9). (**B**) Quantity of phosphorylated Akt (Ser473) (p-Akt) relative to the total Akt (t-Akt), in rat cardiac tissue, with a representative Western blot image shown. 1 mg/kg GDC-0326 significantly decreased Akt phosphorylation when administered prior to ischaemic preconditioning (IPC) or GDC. n = 4 hearts per group. Data is represented as mean ± SEM value. *p < 0.05 assessed using one-way ANOVA with Tukey’s multiple comparisons, ns: nonsignificant differences

We next conducted experiments to assess whether cardioprotection by myosin-targeted inhibitors is independent of the well-established PI3K/AKT cardioprotective pathway (also known as the RISK pathway). We have previously demonstrated that the alpha isoform of PI3K is primarily responsible for cardioprotection via the RISK pathway [20–23]. Therefore, the PI3Kα inhibitor, GDC-0326 was included in IR experiments with IPC and CK-274. As expected, GDC-0326 abrogated IPC-mediated protection, increasing the IS (IPC + GDC: 53 ± 2% vs. IPC alone: 21 ± 3%) (p < 0.05). Interestingly, GDC-0326 also significantly reversed CK-274’s protection (CK-274 + GDC: 62 ± 4% vs. CK-274 alone: 43 ± 2%) (p < 0.05), demonstrating that cardioprotection elicited by CK-274 requires PI3Kα (Fig. 7A).

To confirm the activation of the RISK pathway by both IPC and CK-274, AKT phosphorylation was measured by Western blot analysis of hearts treated *in vivo* with the same treatments as above, in the absence of IR. In these experiments, 2 cycles of IPC did not increase AKT phosphorylation. Nevertheless, GDC-0326 significantly reduced AKT phosphorylation when added to both the IPC and CK-274 groups (both p < 0.05) (Fig. 7B), revealing a role for PI3Kα in mediating CK-274-induced AKT phosphorylation. Full blot images of the western blot can be found in Supplementary Figure S16.

### Effect of CMIs on Histological Changes Following Early Reperfusion

**Fig. 8:**
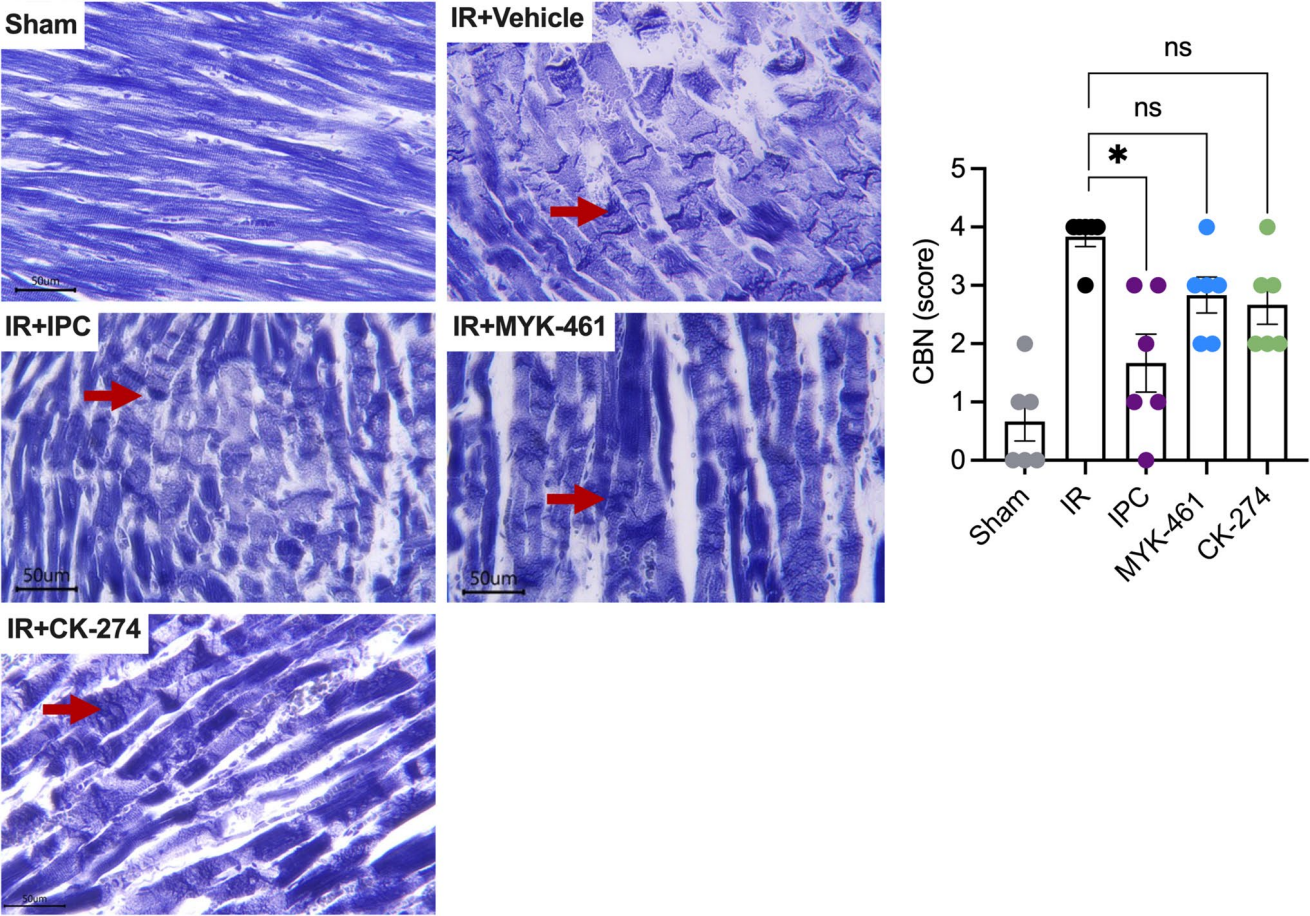
Representative longitudinal sections of PTAH-stained cardiac sections reveal sarcomeres structure and contraction bands following IR. Extensive contraction bands (red arrows) were seen in the IR hearts compared to sham hearts, or IR hearts that had been treated with IPC, MYK-461, or CK-274 hearts. Bar = 50 μm. Legend on the left demonstrated semiquantitative analysis of the extent of contraction bands in PTAH-stained cardiac sections. Data was represented as mean ± SEM, * p < 0.05 assessed using Kruskal–Wallis with Dunn’s post hoc multiple comparisons, n = 6 for each group, ns: non-significant differences

Hypercontracture results in contraction bands necrosis (CBN) (Fig. 8). CBN occur early on during reperfusion and are clearly demarcated by histology with PTAH staining. PTAH staining of sham rat hearts reveals a highly organised structure of normal cardiomyocytes with striations and intercalated discs that are aligned end-to-end and tightly join adjacent cells. However, after IR, CBN is widespread across the tissue section, evident as dark eosinophilic bands spanning the entire width of the sarcoplasm. Some banding was also observed after IR in the groups treated with IPC, 2 mg/kg MYK-461, or 2 mg/kg CK-274 group although it was less distinct than in the IR group. An unbiased qualitative scoring method was developed, to assess the extent of CBN in each heart in a blinded fashion. This demonstrated that IPC (2 ± 0.5) significantly reduced the median CBN score in IR rats compared to the IR alone group (4 ± 0.2, p < 0.05). However, no significant difference was observed between the 2 mg/kg MYK-461 (3 ± 0.3) and 2 mg/kg CK-274 (3 ± 0.3) groups compared to the IR group.

## Discussion

Using an *in vitro* model of cardiomyocyte hypercontracture in this study, we first identified the IC_50_ for CK-274 in this cellular assay as 1 μM, compared to the IC_50_ of 27.4 μM for MYK-461. Subsequently, an *in vivo* study was used to establish a suitable dose at which these drugs could be administered to rats without causing a significant reduction in LV function. Based on these findings, we identified suitable doses that had minimal effect in healthy rats as 2 mg/kg for MYK-461 and CK-274. In a second study, we found that 2 mg/kg MYK-461 reduced LVEF to ~ 45%, and this was significant when compared to rats administered vehicle. However, we confirmed that the effect was transitory and returned to baseline by 24 h. We next showed that 2 mg/kg MYK-461 was cardioprotective, reducing infarct size in rats subject to IR, and did not provide any additional protection to rats that had received IPC. CK-274 was more potent, significantly reducing infarct size at 0.5 mg/kg. It reduced infarct size to a similar extent at 1.0 mg/kg and 2.0 mg/kg. Surprisingly, GDC-0326 abrogated CK-274’s protection, demonstrating that cardioprotection elicited by CK-274 requires PI3Kα. We performed a histological assay of CBN in hearts of rats subject to IR, and banding appeared to be less distinct after IPC, 2 mg/kg MYK-461, or 2 mg/kg CK-274. However, a semi-quantitative analysis of these images was only able to identify a significant effect in IPC hearts.

In our initial *in vitro* experiments, CCCP was used to induce hypercontracture in primary ARVC because it is a potent mitochondrial uncoupler, and is well known to lead to ATP depletion, which causes cellular contracture [30]. The ARVCs were pre-treated with either MYK-461 or CK-274 for 30 min to allow sufficient time for the drugs to be taken up and equilibrate. In this assay, a dose-dependent reduction in hypercontracture onset was seen, with CK-274 being ~ 27 × more potent than MYK-461. However, the *in vitro* IC_50_ values obtained may not directly translate to our *in vivo* dosing as the observed ~ 27X difference in IC_50_ between MYK-461 and CK-274 reflects differences in their potencies under the controlled conditions of the *in vitro* assay. These results were influenced by factors such as assay design and the direct binding affinities of the compounds. For instance, Chuang and colleagues showed that the IC_50_ of CK-274 and MYK-461 in reducing fractional shortening in rats *in vivo* was 7.9 μM and 1.7 μM, respectively. Similarly, CK-274 showed potency towards cardiac myofibrils *in vitro* with an IC_50_ of 1.4 μM [18].

Echocardiography revealed that sufficiently high doses of MYK-461 and CK-274 had effect on LV function even in healthy rats. This aligns with previous findings reported in the literature [15, 18, 35]. The reduction in LVEF was evident more rapidly with CK-274, occurring after just 10 min, compared to MYK-461 which took 30 min. This may reflect their different pharmacokinetics. It was important to note that LV function returned to baseline levels within 24 h. This was due to the fact that CK-274 and MYK-461 has a *t*_1/2_ in rats ranging from 3 to 11 h [18, 36], indicating after this period, the effects of these inhibitors diminish significantly. Nevertheless, 2 mg/kg MYK-461 also led to a significant (though transient) drop in BP, as expected in light of the reduction of LVEF [37].

Notably, CK-274 reduced infarct size at 0.5, 1.0 and 2.0 mg/kg, despite having a significant impact on LVEF only at 2.0 mg/kg. This suggests that CK-274 could be an option for a new therapy for IR injury without causing negative inotropic effects, although the dose must be carefully selected.

While IPC is of limited practical value in the setting of clinical STEMI, due to the fact that it must be administered prior to ischaemia, it nevertheless serves as a valuable experimental tool, consistently inducing robust cardioprotection across various mammalian species [38, 39]. It has been proposed that a multi-target strategy might be required in order to maximise the degree of protection in the context of an AMI [26, 40, 41]. Since CMI act by binding myosin and inhibiting contracture, we hypothesise that their protection would be independent of the RISK pathway, and therefore would be additive with RISK-pathway mediated cardioprotection by IPC. Therefore, we investigated whether CK-274 would be capable of fighting kind of protection that would be additive with IPC. In order to facilitate the detection of additive reduction in infarct size by CK-274 with IPC, an extended ischaemic time (40 min) was used to increase the infarct size caused by IR. Nevertheless, the co-administration of CK-274 alongside IPC did not manifest an additive protective effect. Co-administration of an inhibitor of the PI3Kα arm of the RISK pathway showed that, contrary to our hypothesis, cardioprotection by CK-274 required PI3Kα. We used a specific inhibitor of PI3Kα based on our previous data showing that IPC is completely abrogated by GDC-0326 [23]. This was confirmed by Western blot analysis showing that inhibition of PI3Kα reduced AKT phosphorylation, in either control hearts or in hearts treated with IPC or CK-274. The reason that cardioprotection by CK-274 requires PI3Kα is not clear. There is evidence that PI3Kα activation can increase cardiac contractility [42]. In cardiomyocytes, mechanical stress and alterations in sarcomere function can trigger signalling cascades that include the PI3K pathway. It is possible that, during hypercontracture, the mechanical stress and deformation of the cell activates various signalling pathways, including PI3K/Akt. However, it is more difficult to understand how *inhibition* of contraction with CK-274 might require PI3Kα activity for cardioprotection. However, this phosphorylation of Akt by CK-274 was investigated in non-IR model of healthy animals.

Coronary reperfusion causes cytosolic Ca^2+^ overload. Ca^2+^ overload causes actin to remain persistently bound to myosin, resulting in sarcomere hypercontraction, eventually lead to cardiomyocytes death and CBN [43]. The current study demonstrated the presence of CBN during the early reperfusion phase (at 30 min) in a rat IR model, similar to previous studies, which observed CBN to occur rapidly after reperfusion [44, 45]. Upon reperfusion, CBN represents the initial impairment in the myofibrils, manifesting as a loss of sarcomere integrity [46].

Interestingly, the group that received an IPC intervention prior to IR exhibited a significant reduction in the severity of CBN. This implies that the IPC intervention had a protective effect on the tissue, mitigating the development of severe CBN associated with ischaemic injury. Even though the present data does not elucidate the direct mechanism by which IPC reduces CBN, the cardioprotective signalling cascades by which IPC inhibits reperfusion injury have been extensively studied [19, 21, 47]. Studies have reported that IPC inhibits hypercontracture-induced mechanical injury by reducing cytosolic Ca^2+^ loading and SR-driven Ca^2+^ oscillations in isolated cardiomyocyte [48, 49].

We anticipated a reduction in CBN severity based on the protective effects of MYK-461 and CK-274 in reducing infarct size. However, contrary to the initial hypothesis, in this series of experiments, neither the MYK-461 nor the CK-274 treatment group exhibited a significant reduction in CBN score, when compared to the IR group. It is possible that the mechanisms of action of both CMIs, despite showing promising outcomes in reducing infarct size, may not be as effective in mitigating the development of severe CBN. In addition, challenges in obtaining a quantitative measure of extent of CBN, as well as variability among animals in each group (IR, CK-274, and MYK-461) might influence the ability to achieve statistical significance within these groups. Animals within the treatment groups may exhibit varying responses to the treatment due to inherent genetic and physiological differences [9, 50, 51]. Most likely, the semi-quantitative nature of the scoring procedure, and high heterogeneity of the CBN morphology within each individual heart, resulted in our analysis having high variability, which limited our ability to detect a significant difference. Thus, individual variation could contribute to the variation of CBN scoring within these groups. Unfortunately, there is no more accurate way of measuring CBN in hearts following *in vivo* IR, that we are aware of.

The impact of other co-morbidities, such as ageing, diabetes, or hypertension, remains uncharacterised at present. For effective clinical translation, an ideal and clinically relevant experimental model should involve animals of IR models with co-morbidities such as hypertension, diabetes, or ageing, commonly observed in patients presenting with AMI. This approach would be crucial to understanding how pharmacological manipulation could potentially aid in the management of AMI.

## Conclusion

To the best of our knowledge, this is the first study to investigate the potential of CMIs as cardioprotective agents in the context of myocardial IR injury. This study reveals potential of CMIs as treatments to mitigate the impact of reperfusion-induced hypercontracture, consequently limiting the infarct size following IR. The concept of inhibiting reperfusion-induced hypercontracture that can be beneficial for heart protection was initially derived from prior studies indicating that the use of β-blockers reduces infarct size by lowering inotropy and alleviating ischemia. However, the therapeutic efficacy of this approach has had relatively limited clinical success. Their lack of success is likely attributed to their ability to induce vasoconstriction by unmasking α2-adrenergic vasoconstriction within the ischaemic myocardium [52, 53]. MYK-461 and CK-274 are unlikely to have direct vascular effects, as their actions are specific to cardiac myosin [18]. This specificity makes them suitable candidates for future treatments following an IR injury. Importantly, CK-274 was able to reduce myocardial infarct size at doses that did not significantly affect cardiac ejection fraction. On the other hand, a similar degree of cardioprotection and highly effective reduction in CBN was observed in hearts pre-treated with IPC, which suggests that cardioprotective strategies activating the RISK pathway may limit hypercontracture in addition to limiting infarction.

A limitation of the present study is that it used an acute and short-duration of MI model *in vivo* with an open chest, hence presenting limitations for cardiac function studies. Consideration might be given to employing a chronic model of MI or a closed-chest *in vivo* approach, which could enable cardiac function studies using techniques like 2D echocardiography. It would be interesting to investigate whether these CMIs could reduce infarct size in an MI model without adversely affecting cardiac function, as preserving cardiac function is crucial. It is important to also consider the risk of hypotension, pulmonary oedema and/or cardiogenic shock. This approach could further establish a link between the benefits of reperfusion-induced hypercontracture by these CMIs and the reduction in infarct size.

## Supplementary Information

Below is the link to the electronic supplementary material.

Supplementary file1 (DOCX 29508 KB)

## Data Availability

All data and material are available upon reasonable request.

## Abbreviations

BDM: 2,3-Butanedione monoxime
CBN: Contraction band necrosis
CCCP: Carbonyl cyanide m-chlorophenylhydrazone
CMIs: Cardiac myosin-targeted inhibitors
IPC: Ischaemic preconditioning
