# Supplementary material for: Novel Selective Cardiac Myosin-Targeted Inhibitors Alleviate Myocardial Ischaemia–Reperfusion Injury
Source: Cardiovasc Drugs Ther. 2025 Jan 4;40(1):39–52. doi: 10.1007/s10557-024-07663-0 (PMC12872633; doi:10.1007/s10557-024-07663-0)
Supplement: Supplementary file 1 — Supplementary file1 (DOCX 29508 KB) [file 10557_2024_7663_MOESM1_ESM.docx]

Supplementary Materials

**Novel selective cardiac myosin-targeted inhibitors alleviate myocardial ischaemia-reperfusion injury**

Nur Liyana Mohammed Yusof^1,2,^ Derek M Yellon^1^ and Sean M Davidson^1*^

^1^ The Hatter Cardiovascular Institute, University College London, 67 Chenies Mews, London WC1E 6HX, United Kingdom

^2^ Department of Pharmacology, Faculty of Medicine, Universiti Kebangsaan Malaysia, Jalan Yaacob Latif, Bandar Tun Razak, 56000 Cheras, Kuala Lumpur, Malaysia

**Institution where the works has been done/ mailing address:**

The Hatter Cardiovascular Institute, University College London, 67 Chenies Mews, London WC1E 6HX, United Kingdom

***Corresponding author:** s.davidson@ucl.ac.uk

**Methods**

**Experimental protocols (2D Echocardiography)**

**Study 1**

Real-time ultrasound images were acquired at baseline and 30 min post-IP injection of several doses of MYK-461, as illustrated in **Figure S1** below.


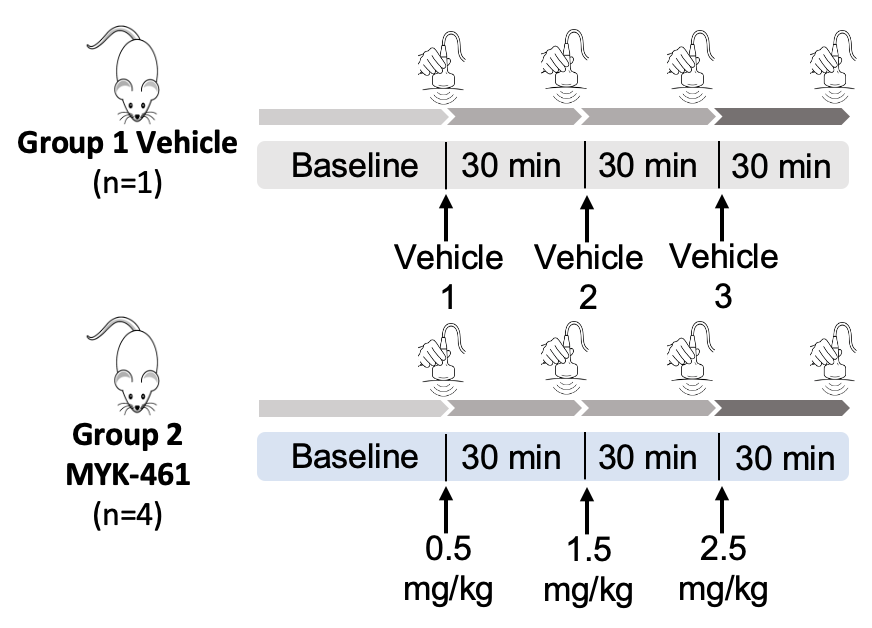


***Figure S1: Experimental design of ultrasound scanning.***

*LVEF was measured at baseline and every 30 min post-injection of MYK-461 via IP. Three different doses of MYK-461 were injected into the same rat (cumulative dose of 4.5 mg/kg) with a 30 min time difference between the doses. 90% corn oil and 10% DMSO as a vehicle.*

**Study 2**

A second study was conducted to acquire real-time ultrasound images at baseline throughout 2 h, and 24 h after the IP injection of MYK-461 at a dose of 2 mg/kg, as depicted in **Figure S2** below. Since this study focused on the recovery phase, invasive BP monitoring was not performed.


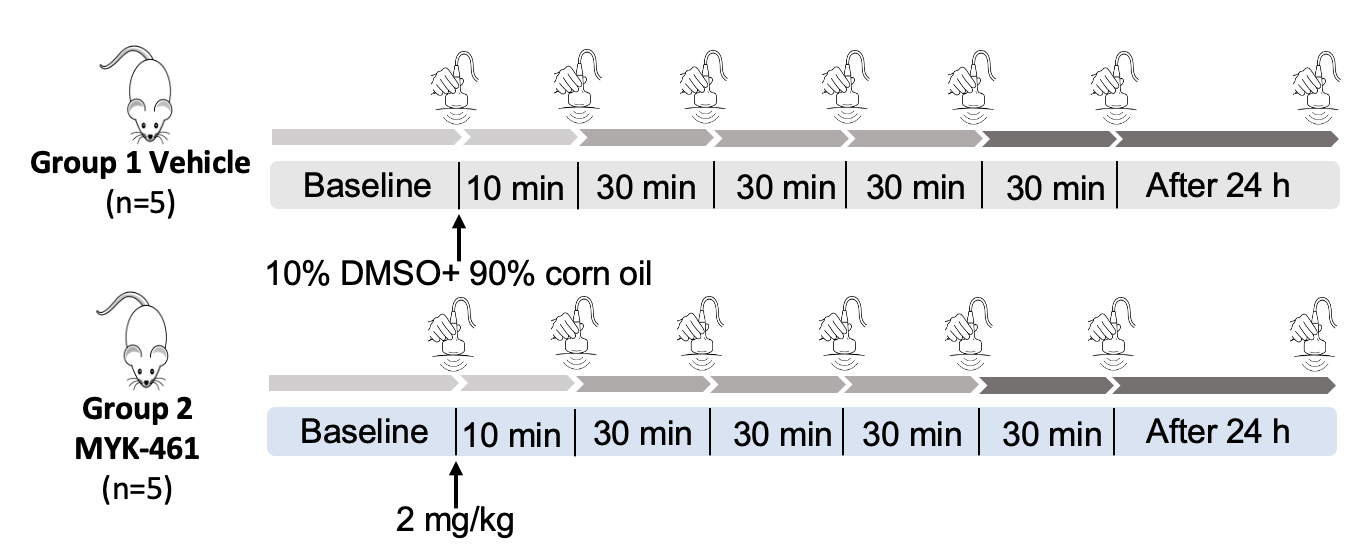


Figure S2: Experimental design of ultrasound scanning.

*LVEF was measured at baseline, within 2 h, and 24 h post-injection of 2 mg/kg MYK-461 via IP.*

**Study 3**

A separate set of experiments was performed to obtain real-time ultrasound images at baseline, after 10 min, and every 30 min following IP injection of CK-274 at doses of 0.5 mg/kg, 1 mg/kg, and 2 mg/kg, as well as MYK-461 at a dose of 2 mg/kg, over a 3-h duration. **Figure S3 below** provides a visual representation of the experimental timeline. Additionally, BP measurements were obtained using a fluid-filled catheter inserted into the right carotid artery and connected to the PowerLab system for continuous monitoring.


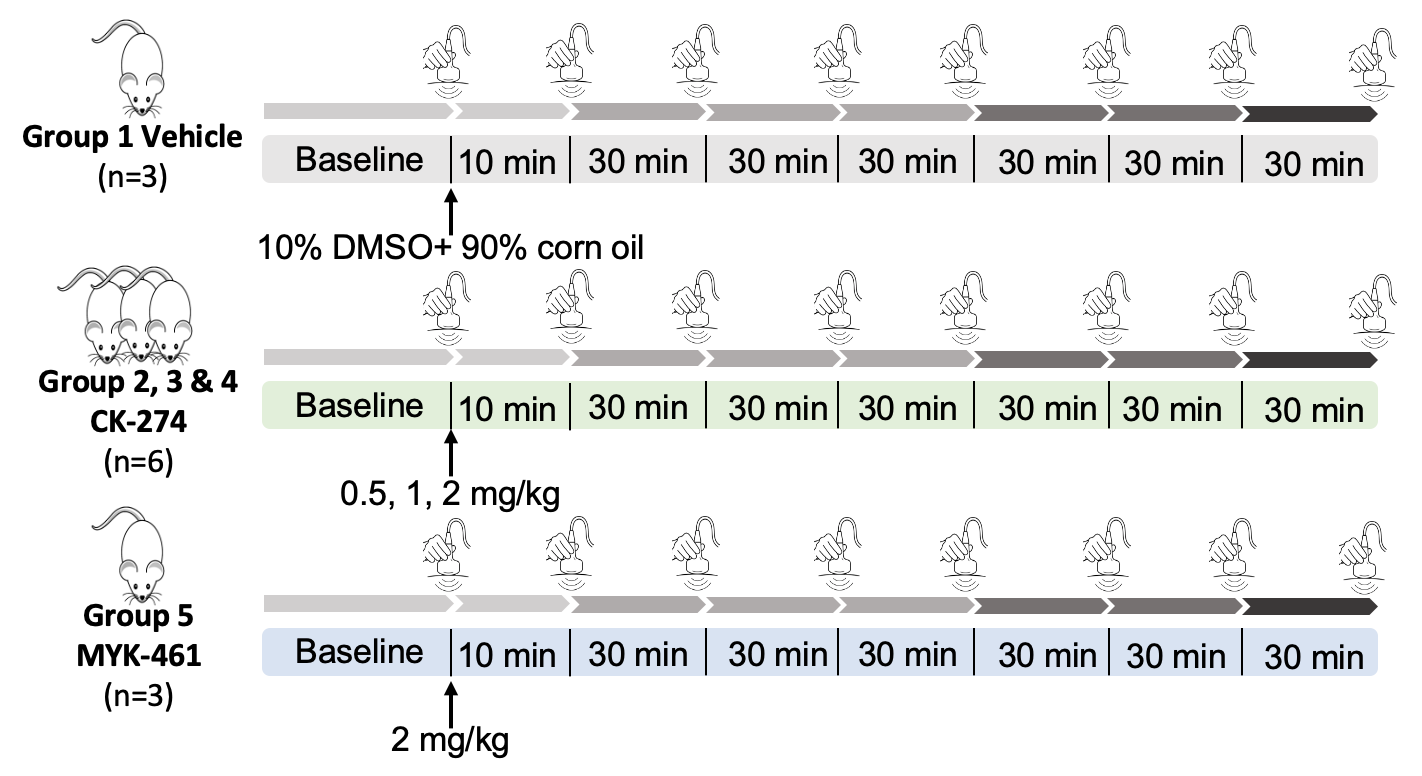


***Figure S3: Experimental design of ultrasound scanning.***

*LVEF was obtained at baseline, 10 min, and every 30 min post-injection of 3 different doses of CK-274 (0.5, 1, and 2 mg/kg) individually as well as 2 mg/kg of MYK-461 via IP.*

**Induction of myocardial ischaemia and reperfusion in vivo and infarct size measurement**

Outlines for the study protocols for the infarct size measurement was illustrated as follows:

**Study 1**


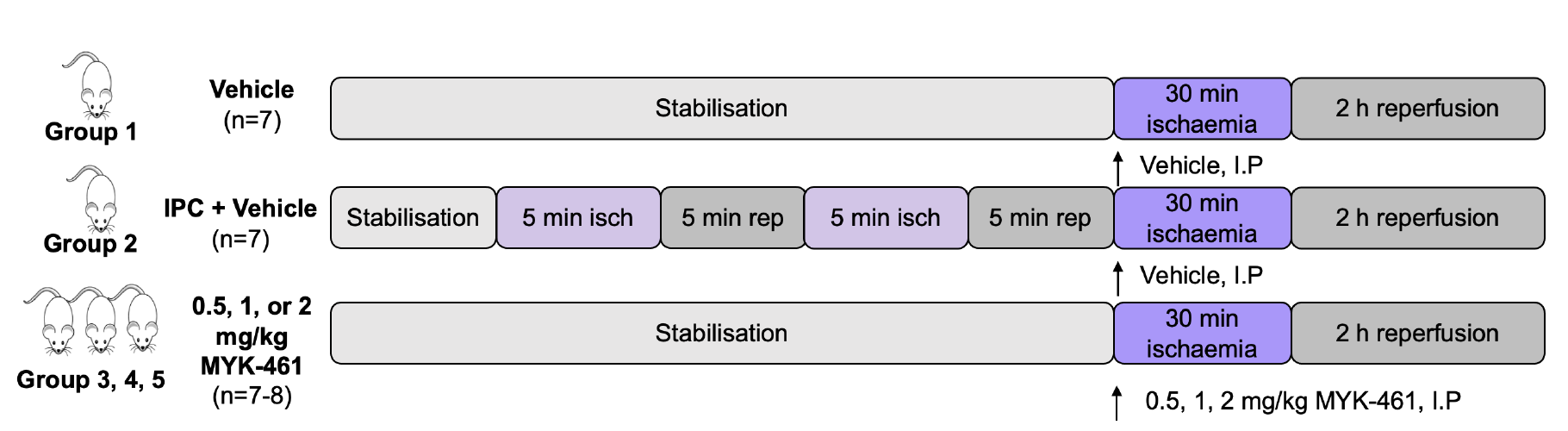


**Figure S4: Experimental protocol for IR non-recovery in vivo with varying dosages of MYK-461 treatment and the IPC protocol.**

After stabilisation, a two-cycle IPC of 5 min ischaemia and 5 min reperfusion was applied prior to 30 min ischaemia and 2 h reperfusion. Vehicle, 0.5, 1, or 2 mg/kg of MYK-461 were administered via IP bolus 30 min prior to the onset of reperfusion, isch: ischaemia, rep: reperfusion.

**Study 2**


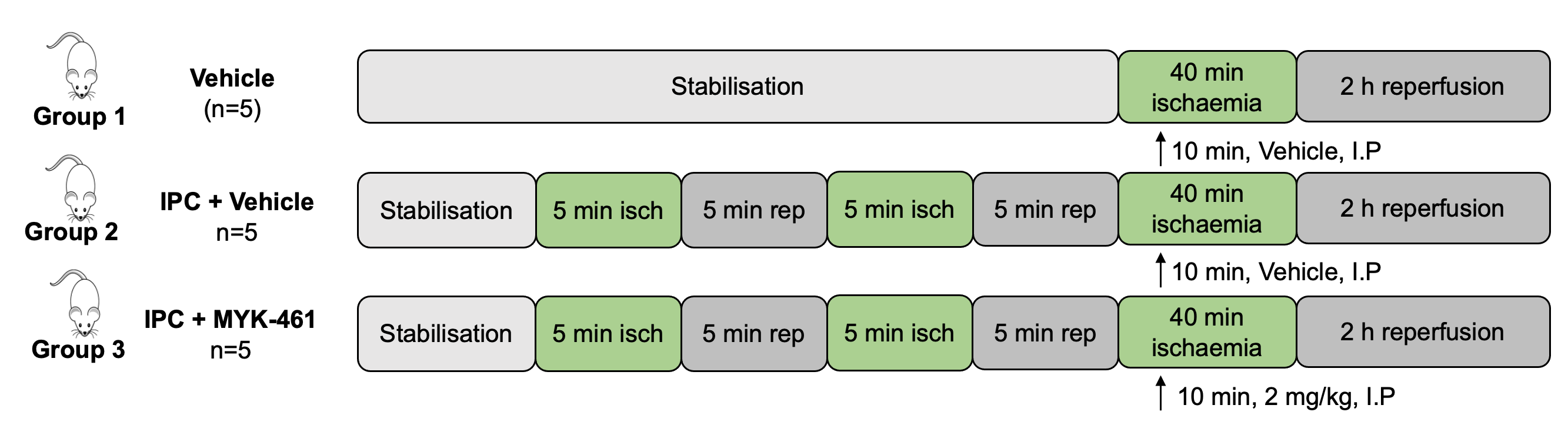


**Figure S5: Experimental protocol for IR non recovery in vivo combining MYK-461 treatment and IPC protocol.**

After stabilisation, a two-cycle IPC of 5 min ischaemia and 5 min reperfusion was applied prior to 30 min ischaemia and 2 h reperfusion. Vehicle or 2 mg/kg of MYK-461 were administered via IP bolus 30 min prior to the onset of reperfusion, isch: ischaemia, rep: reperfusion.

**Study 3**


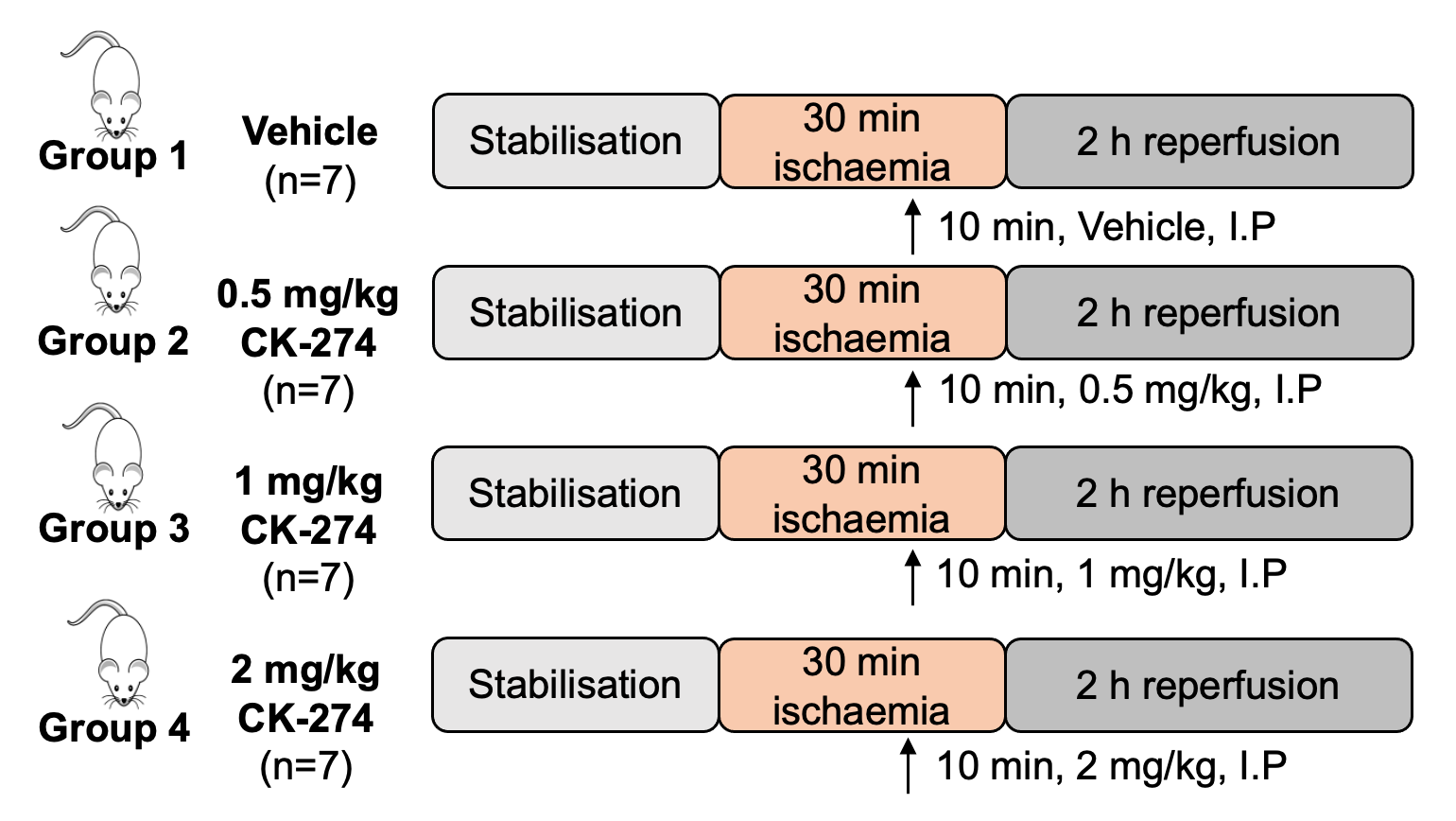


**Figure S6: Experimental protocol for IR non-recovery in vivo with varying dosages of MYK-461.**

After stabilisation, LAD was ligated for 30 min and followed by 2 h reperfusion. Vehicle or 0.5, 1 or 2 mg/kg of CK-274 were administered via IP bolus 10 min prior to the onset of reperfusion.

**Study 4**


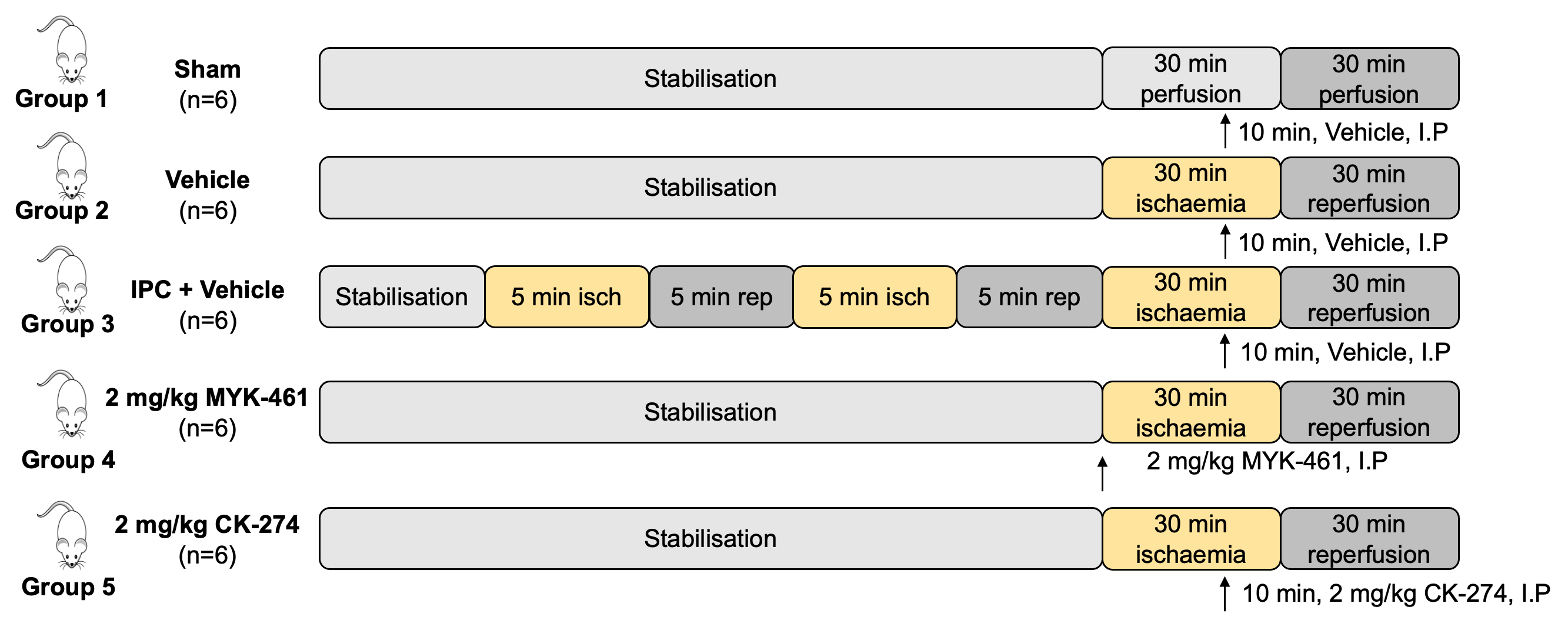


**Figure S7: Experimental protocol for IR non-recovery in vivo with varying dosages of MYK-461.**

After stabilisation, the LAD coronary artery was ligated for 30 min, followed by 30 min of reperfusion. Vehicle, 2 mg/kg of MYK-461 or CK-274 were administered via IP bolus 10 / 30 min prior to the onset of reperfusion. IPC served as a positive control. At the end of the 30 min reperfusion, the hearts were excised for histology assessment.

**Western blot**

Outlines for the study protocols for the western blot was illustrated as follows:

**
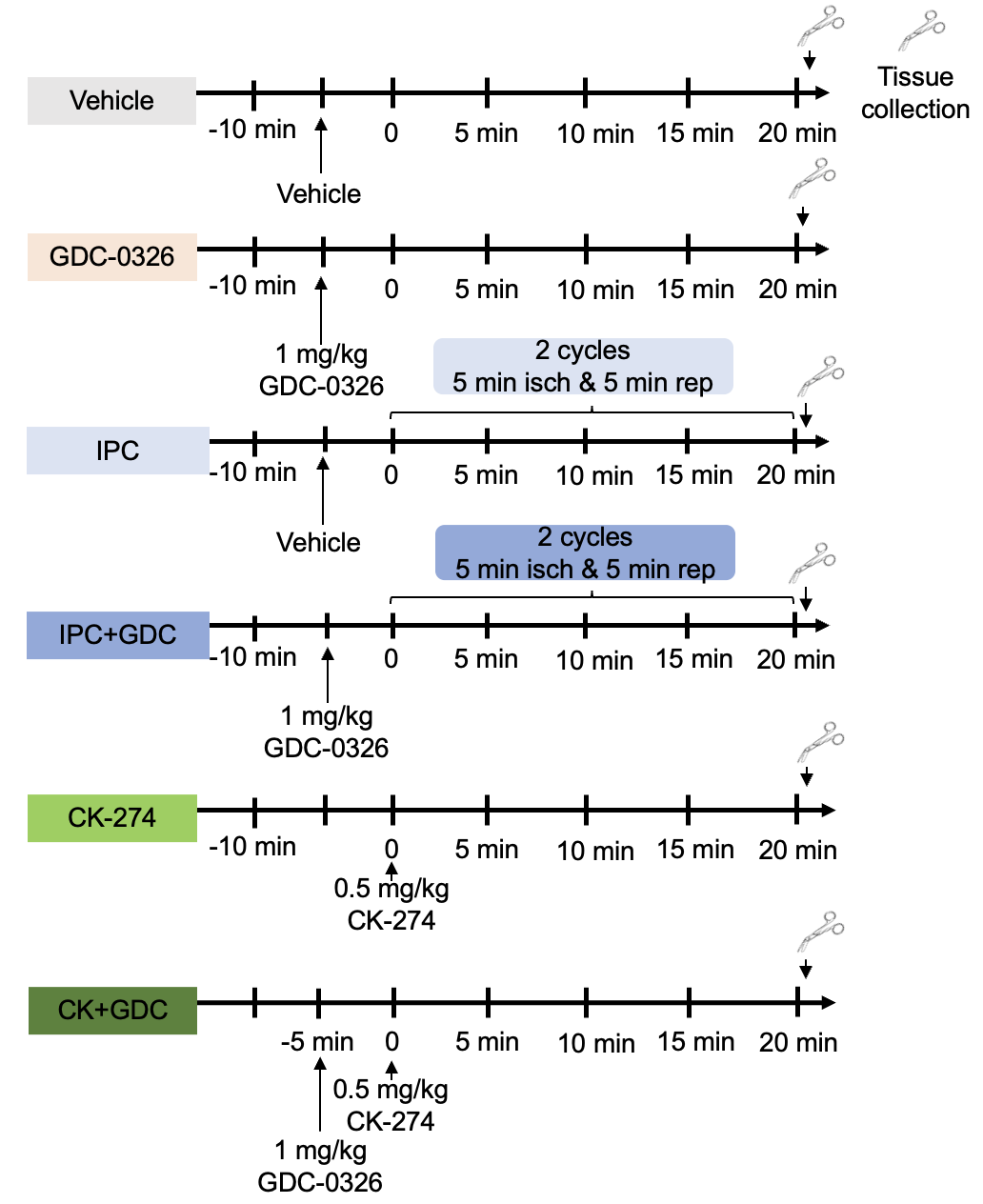
**

**Figure S8: Illustration of the experimental protocols.**

Arrow indicates time of IP administration of the PI3Kα inhibitor, GDC-0326 (1 mg/kg) and CK-274 (0.5 mg/kg).

**Histology study (Experimental protocol)**
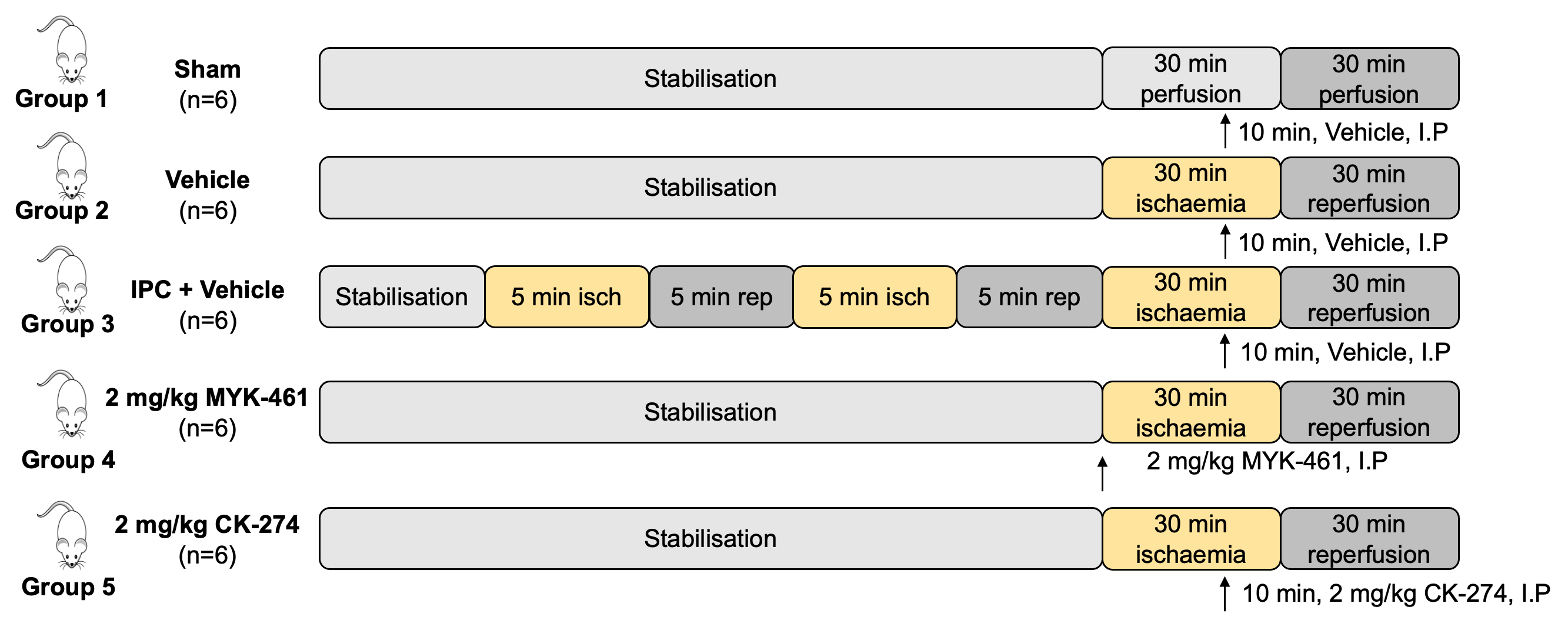


Figure S9: Experimental protocol for IR non-recovery in vivo for histology study.

*After stabilisation, the LAD coronary artery was ligated for 30 min, followed by 30 min of reperfusion. Vehicle, 2 mg/kg of MYK-461 or CK-274 were administered via IP bolus 10 / 30 min prior to the onset of reperfusion. IPC served as a positive control. At the end of the 30 min reperfusion, the hearts were excised for histology assessment.*

**CBN scoring**

All the images of stained slides were taken using an inverted microscope (GXM) at 20X magnification with GXCAM-U3PRO-6.3 software. For each slide, a total of five images were captured, and the image acquisition process began at the apex (the lowest point or tip) of the heart section. This was to ensure that the image acquisition was focused on the AAR, where the hypercontracted cardiomyocytes were usually present. Under a light microscope, CBN is characterised by the appearance of hypercontracted bands within muscle fibres. These bands are highly contracted areas that stand out from the surrounding tissue. The hypercontracted bands also exhibit cross-striations, which are indicated by dark bands when viewed under the microscope. To analyse this CBN, each stained slide was coded by a blind operator. As illustrated in **Figure S6** below, the grid function on ImageJ was employed to calculate the percentage of positive CBN staining over the total number of grids. This method allowed for a quantitative assessment of CBN distribution in the histological images. The percentage of CBN was calculated using the formulas as shown below.


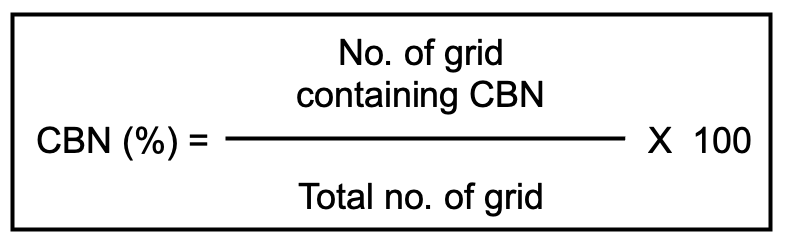


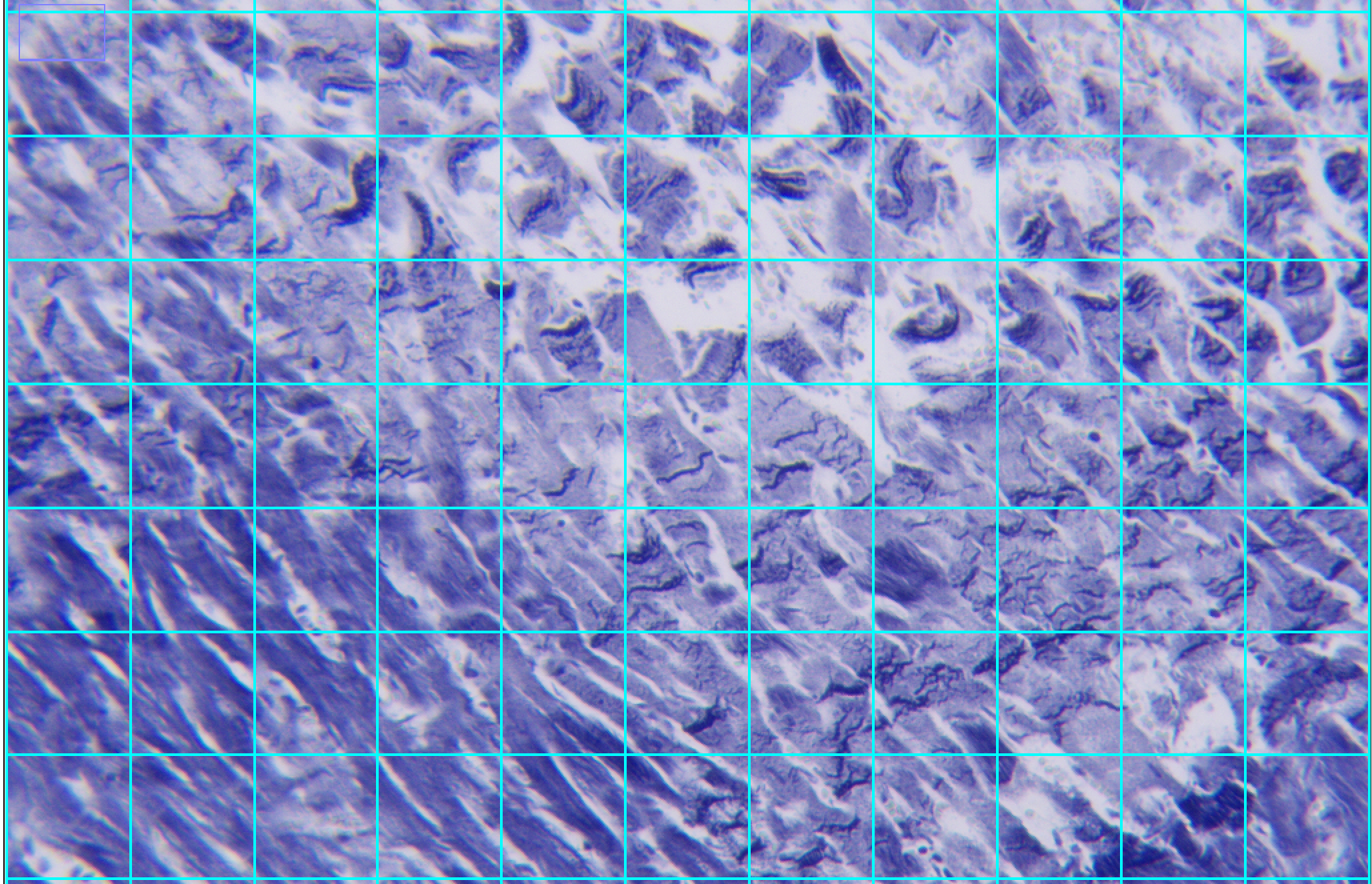


Figure S10: Representative histological image with grid overlay.

*The histological image demonstrated CBN under PTAH staining (20x). A grid overlay, generated using ImageJ, has been applied for semi-quantification analysis of CBN. The grid facilitates the quantification of positive staining, particularly the darker eosinophilic bands representing contraction band necrosis (CBN) distribution. Each grid unit containing CBN was individually counted. The total number of grids containing CBN was then calculated as a percentage of the total number of grids covering the entire area.*

To semi-quantify the CBN in each group, ordinal scores were used based on the distribution of CBN (in %). A 5-class scoring system was used, as shown in **Table 1** and **Figure S7** below. The scoring process was conducted blindly.

Table 1: Ordinal scores based on distribution of CBN.

| Score | CBN (%) |
| --- | --- |
| 0 | 0 |
| 1 | < 25 |
| 2 | 26-50 |
| 3 | 51-75 |
| 4 | 76-100 |


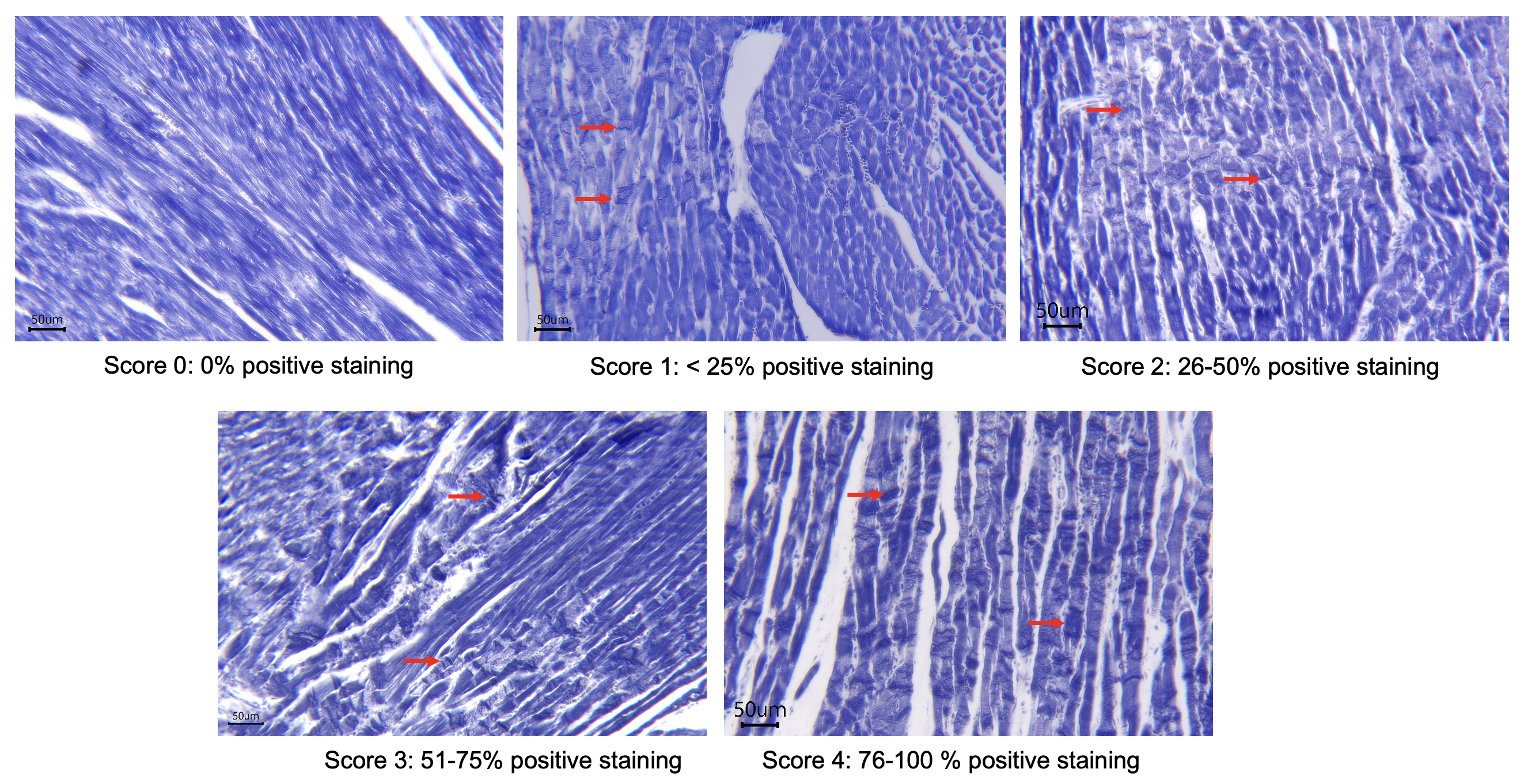


***Figure S11: Representative images of heart samples stained with PTAH exhibiting widely distribution of CBN (red arrows).***

*An ordinal scoring system was used based on the estimated percentage of CBN on each slide; in this case, a score of 0 to 4 was assigned.*

**Results**


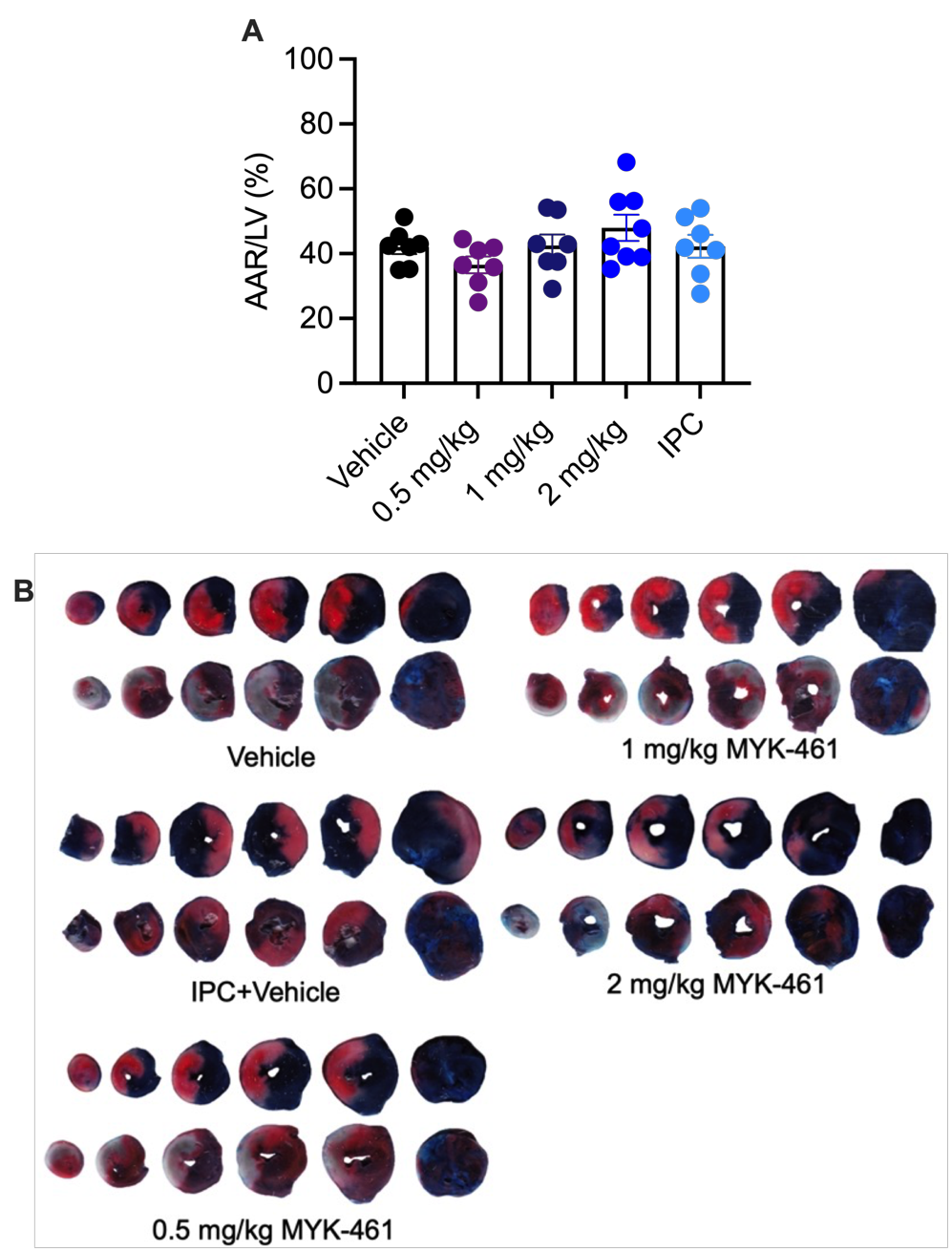


***Figure S12****: The AAR (A) was presented as a percentage of the LV while representative consecutive segments of rat hearts from apex to base (B) stained with Evans blue and TTC from each experimental group in* ***Figure 4.*** *The slices underwent two scans each, where the upper slices were stained with Evan’s blue used for AAR analysis, while the lower slices were then stained with TTC staining for infarct size assessment. Under Evan’s blue staining, the red area represents the AAR, while the infarcted myocardium is visualised as greyish to white under the TTC staining, while the viable and AAR myocardium is stained red.*


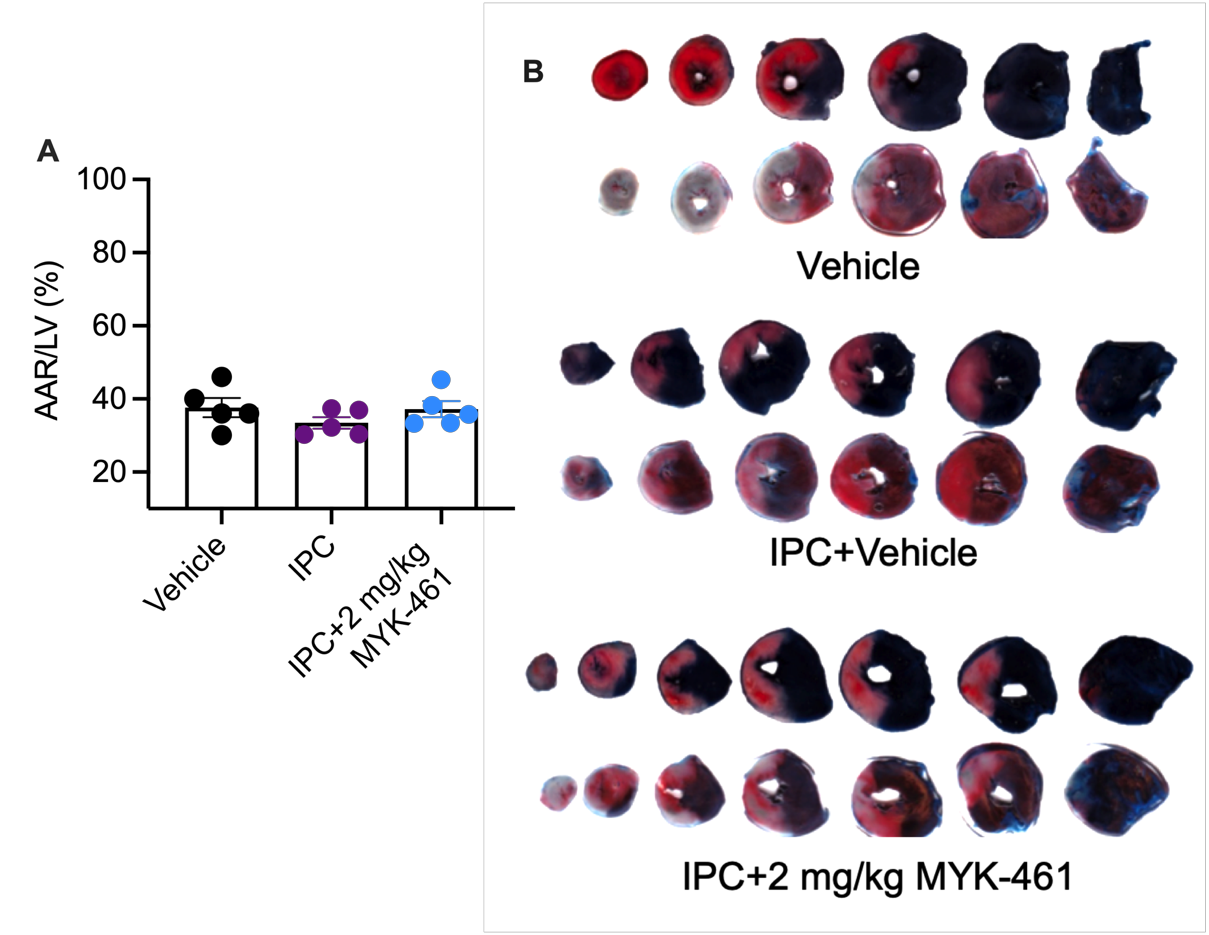


***Figure S13****: The AAR (A) was presented as a percentage of the LV while representative consecutive segments of rat hearts from apex to base (B) stained with Evans blue and TTC from each experimental group in* ***Figure 5.***


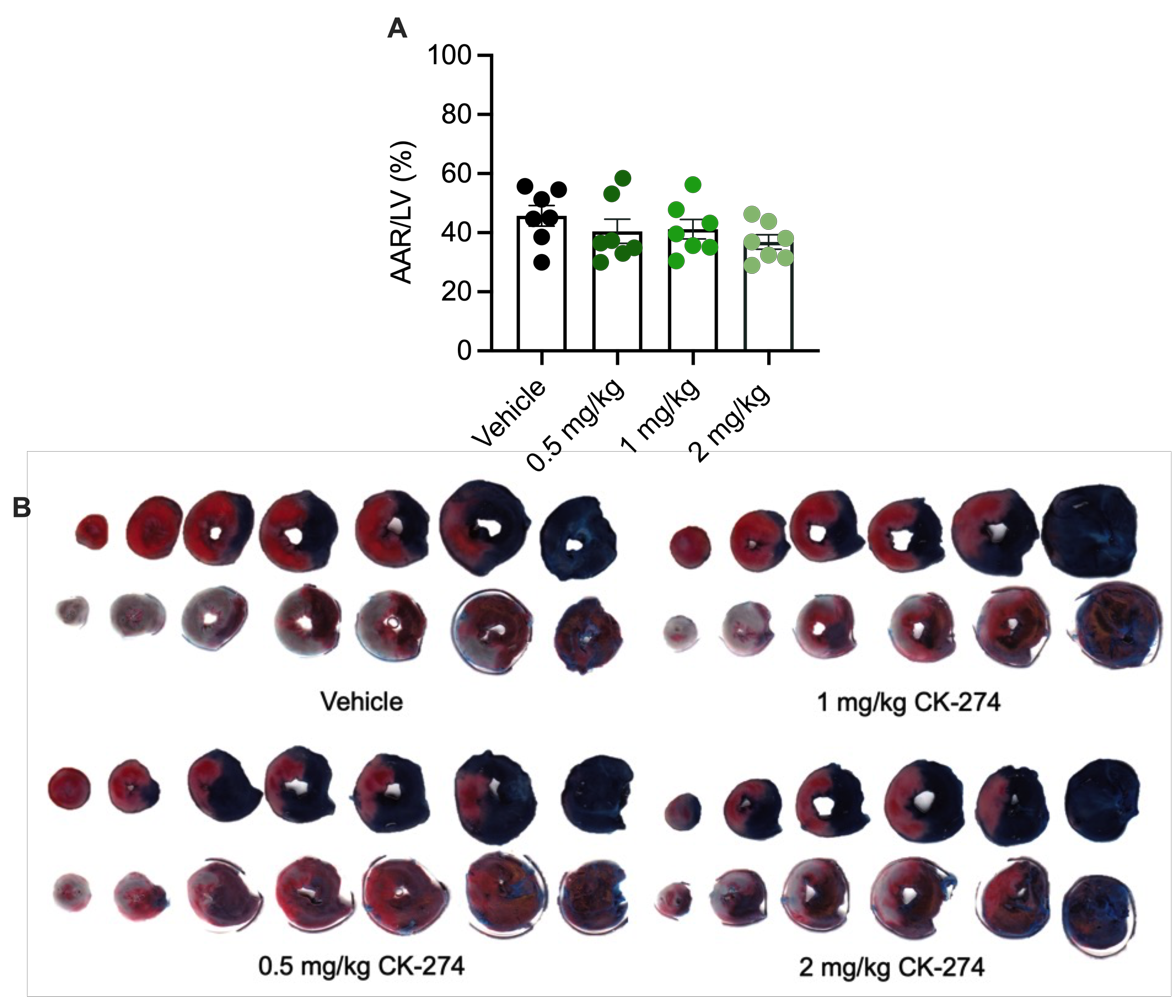


***Figure S14****: The AAR (A) was presented as a percentage of the LV while representative consecutive segments of rat hearts from apex to base (B) stained with Evans blue and TTC from each experimental group in* ***Figure 6.***


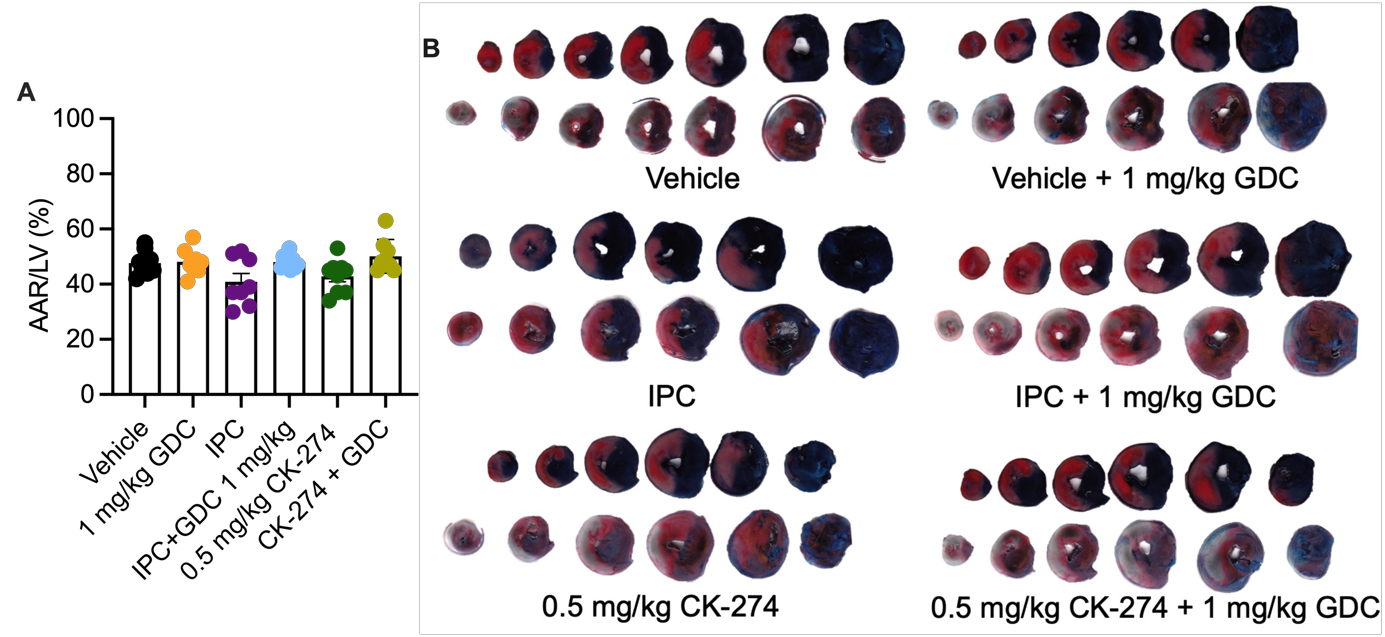


***Figure S15****: The AAR (A) was presented as a percentage of the LV while representative consecutive segments of rat hearts from apex to base (B) stained with Evans blue and TTC from each experimental group in* ***Figure 7A.***

***Figure S16: Full blot images of the western blot for all samples.***

**For membrane 1**

List of samples from left:

Vehicle (sample1), CK-274+GDC (sample1), IPC (sample1), GDC(sample1), CK-274 (sample1), IPC+GDC (sample1), Vehicle (sample2), GDC (sample 2), IPC (sample 2), IPC+GDC (sample 2), CK-274 (sample 2), CK-274+GDC (sample 2)].

**For membrane 2**

List of samples from left:

IPC+GDC (Sample 3), GDC (sample 3) , CK-274 (sample 3), IPC (sample 3), Vehicle (sample 3), CK-274+GDC (sample 3), Vehicle (sample 4), GDC (sample 4), IPC (sample 4), IPC+GDC (sample 4), CK-274 (sample 4), CK-274+GDC (sample 4)
